# Sprayable Hydrogel Dressings in Wound-Healing Applications

**DOI:** 10.3390/bioengineering13060618

**Published:** 2026-05-25

**Authors:** Lei Nie, Yuanyuan Lu, Wei Guo

**Affiliations:** College of Life Sciences, Xinyang Normal University, Xinyang 464000, China; lyuanyuan0211@163.com (Y.L.); weiguo@xynu.edu.cn (W.G.)

**Keywords:** hydrogels, wound dressing, sprayability, wound healing

## Abstract

With an increased number of chronic wounds and accidents worldwide, the need for advanced wound care approaches has been urgent. In this regard, sprayable hydrogel dressings have emerged as an innovative biomaterial due to their unique rheological properties, minimally invasive operation capabilities, excellent adaptability to irregular surfaces, and in situ rapid gelation. This review focused on elaborating the main materials used to construct sprayable hydrogels, including natural polymers and synthetic polymers, and discussing their respective molecular structures, physicochemical properties, advantages, and challenges in formulation design. This review also explored the properties of sprayable hydrogels, including sprayability, adhesion performance, mechanical strength, moisture absorption, breathability, biocompatibility, and degradability. The mechanisms of their controllable gelation through chemical crosslinking and physical crosslinking strategies were analyzed. Subsequently, the applications of sprayable hydrogels in wound areas, including diabetic wounds, infected wounds, postoperative adhesions, burn wounds, and joint wounds, were comprehensively reviewed. The challenges and future developments in wound healing were clarified to provide valuable references for promoting interdisciplinary research and the clinical translation of sprayable hydrogels.

## 1. Introduction

The skin serves as the first physiological barrier of the human body, and its integrity is crucial for preventing pathogen invasion and maintaining internal homeostasis [1]. The repair of acute and chronic wounds, such as burns, diabetic ulcers, and surgical trauma, is a complex and dynamic physiological process involving multiple stages, including the hemostasis phase, the inflammatory phase, the proliferative phase, and the remodeling phase [2]. An ideal wound dressing should not only protect the wound and prevent infection, but also actively promote this healing process [3]. Traditional dressings such as gauze and sponge often have difficulty adhering closely to irregular wounds, are prone to causing secondary damage during replacement, and cannot provide a moist healing environment [4]. There are still significant challenges in managing complex wounds with high exudate and high infection risk.

The dual-syringe injectable hydrogels represent a well-established clinical tool. They offer several advantages including separate storage of reactive components which prevents premature gelation, the mixture can be pre-cured for a few seconds to achieve a desired viscosity, and they can effectively fill deep or cavity wounds. However, for large, irregular, or highly dynamic wound surfaces, such as extensive burns, abrasions on moving joints, or battlefield wounds, point injection often fails to provide uniform coverage and may require multiple applications. Sprayable hydrogels address this gap by delivering a fine mist of precursor solution that rapidly forms a conformal, adhesive film over any topography, including concave or convex geometries. Furthermore, sprayable hydrogels are uniquely suited for ultra-thin coatings, temporary fixation of skin grafts, and retention of co-injected therapeutic scaffolds or cells. Hydrogels, due to their three-dimensional network structure with high water content, can simulate the extracellular matrix (ECM) and create an ideal moist healing environment for wounds [5,6]. Hydrogels have become a highly promising material in the field of modern wound care. However, traditional hydrogel sheets or films often suffer from poor adhesion, incomplete coverage, and inconvenient use when applied to deep, narrow, or highly uneven wounds [7]. For small-scale wounds, there is a well-established clinical system for directly applying water gel precursor solutions or tissue adhesives using syringes, including double-syringe systems [8]. Some of these products have been approved by the FDA and are widely used for surgical hemostasis, tissue sealing, and wound dressings. It is important to recognize that liquid injectable hydrogels (e.g., dual-syringe systems) can also cover irregular wounds by flowing into contours, particularly when the wound is kept upright. They remain the preferred choice for deep cavity filling and for delivering cells or sensitive biologics, as the spraying process may compromise bioactivity due to shear forces or crosslinking chemistry. Sprayable hydrogels do not outperform injectables in drug delivery or mechanical strength. Instead, their genuine niche advantages include uniform coverage of very large or geometrically complex surfaces and application on wounds that cannot be positioned upright, where a liquid injectable would run off. In addition, the formation of ultra-thin, breathable layers could prevent maceration in superficial wounds or serve as temporary adhesives for skin grafts [9].

The fluid properties of the sprayable hydrogel could seamlessly cover any irregular, deep, or dynamic areas, such as at joints wounds, forming a physical barrier without the need for surgical suturing or fixation, thus achieving true minimally invasive application [10]. Furthermore, the sprayable hydrogel has been considered an ideal carrier for active molecules, including antibiotics, growth factors, antioxidants, and other drugs [11,12]. Then, these molecules could directly act on the wound surface, precisely regulating the healing microenvironment and accelerating tissue regeneration. The spraying process is simple and quick and is particularly suitable for emergencies, such as in emergency clinics. It can immediately form a protective layer on the wound surface, effectively stop bleeding, seal the wound, and prevent bacterial infection [13].

Although sprayable hydrogels show great potential for wound healing, they have certain drawbacks compared to other forms of hydrogels, such as preformed hydrogel sheets and injectable hydrogels. The precursor solution of sprayable hydrogels typically requires low viscosity, which can lead to insufficient mechanical strength after in situ gelation. Additionally, their tissue adhesion and in situ retention capabilities are limited, making them prone to detachment and thereby compromising continuous therapeutic effects. The spray dosage and coverage uniformity are significantly influenced by device design, solution rheological properties, and operational techniques, posing challenges for accurate drug delivery. Therefore, the effectiveness of sprayable hydrogels in practical applications largely depends on the choice of materials, cross-linking mechanisms, and the thoughtful design of the spraying device [10,13]. In recent years, numerous high-quality reviews have emerged in the field of sprayable hydrogels, providing a solid foundation for ongoing research. Although these reviews address various application scenarios and cross-linking mechanisms, their discussions on the specific application of sprayable hydrogels in wound healing remain relatively scattered and lack comprehensive coverage. Currently, there are few reviews dedicated exclusively to the use of sprayable hydrogels in wound healing, with the most existing literature focusing on a single material system or a specific cross-linking strategy [10]. Based on this, this review is conducted based on the framework of “clinical needs → materials and design strategies → ideal performance”. The goal is to systematically explore the relationships among material selection, performance optimization, and wound-healing efficacy, while providing a comprehensive overview of the latest advances in sprayable hydrogels for wound-healing applications. This review aims to serve as a valuable reference for researchers in the field. The review also discussed the materials used to construct sprayable hydrogels, highlighting both natural and synthetic polymers and their characteristics for the design of wound dressings. The review focused on summarizing the properties that determine the effect of their clinical applications, such as rheology and sprayability, wet tissue adhesion, mechanical strength, antibacterial ability, biodegradability, and drug-controlled-release behavior, and elaborating on various crosslinking strategies for achieving in situ rapid gelation, such as Schiff base reaction, Michael addition, and photo-crosslinking. The specific application cases and therapeutic effects of these materials in the management of acute wounds (such as burns) and chronic wounds (such as diabetic ulcers) were summarized. The goal is to systematically explore the relationships among material selection, performance optimization, and wound-healing efficacy, with a particular emphasis on clinical scenarios where sprayable hydrogels outperform conventional dressings or injectable hydrogels, namely, large irregular wounds, dynamic joint surfaces, hard-to-reach anatomic sites, thin conformal coatings, skin graft fixation, and as retention matrices for injectable therapeutics.

The short-term goal of this review is to provide a systematic overview of the physicochemical properties and manufacturing techniques of sprayable hydrogels and to highlight their already proven clinical applications, such as hemostasis in acute trauma, infection control in partial-thickness burns, and prevention of post-surgical adhesions. The long-term goal is to identify current hurdles, including insufficient mechanical strength after gelation, limited retention on highly exuding wounds, and the lack of standardized spraying devices, and to offer design guidelines for future developments. By clearly distinguishing what is already feasible from what requires further research, we aim to help biomaterial designers focus their efforts on the most pressing unmet clinical needs where sprayable hydrogels offer a genuine advantage over injectable or pre-formed dressings. The goal of this review is not to claim optimization or proven efficacy, but rather to systematically map the relationships between material selection, resulting physical properties, and reported biological outcomes. By identifying which material choices lead to which physical behaviors, we aim to help researchers design sprayable hydrogels for clinical scenarios where no satisfactory option currently exists and to inspire future developments for other challenging applications.

## 2. Sprayable Hydrogel Dressings

**Figure 1:**
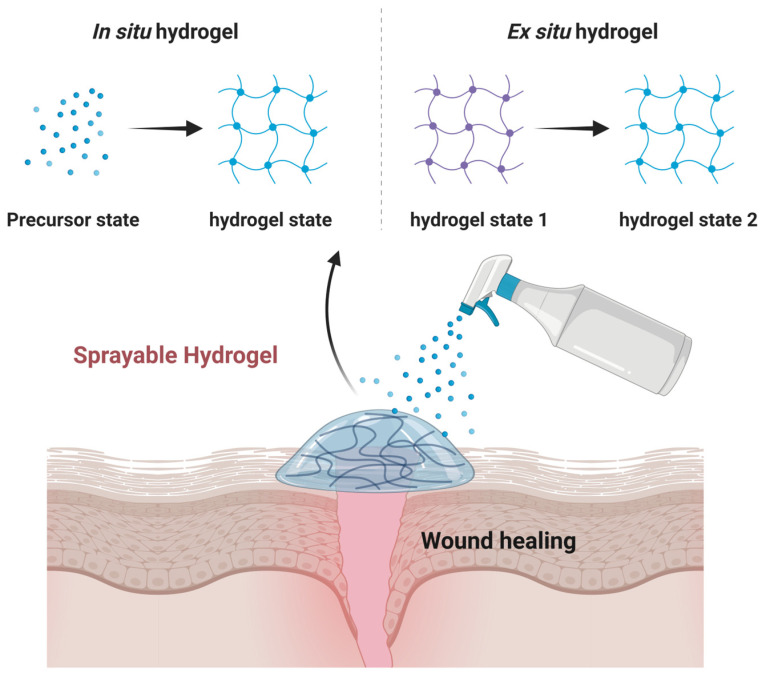
Sprayable hydrogel dressings for wound-healing applications can be classified into in situ and ex situ hydrogels.

Sprayable hydrogel refers to a hydrogel material with specific rheological properties that can be uniformly applied to the substrate surface as droplets or via atomization with spraying equipment and then solidified to form a polymer coating with a three-dimensional (3D) network structure under certain conditions (Figure 1) [13]. In this process, the material exhibits shear-thinning properties and can be transported in a sol state and then quickly formed into a gel state by physical or chemical cross-linking in situ [14]. The sprayable hydrogel with adhesive properties can strongly adhere to wet surfaces and shows good biocompatibility. In addition to tissue adhesion, material design and mechanical properties are also critical to enabling this unique property to be sprayed [15].

Sprayable hydrogels are usually formed by physical or chemical crosslinking of hydrophilic polymers and exhibit good biocompatibility, degradability, and water absorption and retention [16,17,18]. Various crosslinking methods have been explored to render the gel sprayable, including ultraviolet curing, thermal response gluing, and chemical crosslinking (Table 1) [19,20,21,22]. These methods allow the hydrogel to quickly solidify under specific conditions after spraying, forming a stable gel layer. The rapid curing of sprayable hydrogels through photoinitiated free radical polymerization can precisely control the gelation process and adjust the mechanical properties. However, the penetration depth of light in tissues is limited, which restricts its application in deep tissues. Chemical crosslinking does not rely on external stimuli and spontaneously forms a covalent network after mixing, with high gel strength. Among them, the dynamic Schiff base bond reaction is mild, while traditional crosslinking agents (such as glutaraldehyde) have potential toxicity. It has strict requirements for the operation time, and the immediate mixing design during the spraying process is particularly crucial. Physical crosslinking utilizes the self-assembly behavior of temperature- or pH-responsive polymers, without the need for crosslinking agents or photoinitiators, and has the best biocompatibility. However, the mechanical strength of the physical crosslink network is weaker, the gelation speed is slower, and it is prone to structural disintegration. As a novel biomaterial, sprayable hydrogels have attracted significant attention in medicine, bioengineering, and material science in recent years [11]. Sprayable hydrogel dressings are among its common application forms and have been widely used in wound care, including burns, postoperative infections, and diabetic wounds. The sprayable hydrogel dressing creates a moist and breathable environment for the wound, promoting healing. In particular, sprayable hydrogel dressings could offer unique benefits for treating complex wounds and hard-to-reach areas when combined with encapsulated drugs, growth factors, and cells [23].

Sprayable hydrogel dressings possess good properties characteristic of traditional hydrogel materials, including biocompatibility, antibacterial efficacy, degradability, flexibility, and lubricity. Simultaneously, the sprayable design enables the hydrogel dressing to effectively and evenly cover complex and irregular wound surfaces [24]. Additionally, it exhibits adhesive properties and can be precisely applied to frequently mobilized wound sites (such as joints), thereby mitigating the risk of wound infection caused by dressing displacement [25]. Sprayable hydrogel dressings expedite healing by rapidly polymerizing into colloidal forms via a simple spraying technique on the wound surface. Moreover, this approach obviates the need for cumbersome dressing procedures or immobilization steps associated with conventional wound dressings. The convenience and immediacy of sprayable hydrogel dressings are particularly advantageous in emergency wound treatment scenarios, not only facilitating the use of medical personnel but also mitigating patient discomfort and inconvenience [26]. Moreover, sprayable hydrogel wound dressings are suitable for a wide range of wounds, such as traumatic incisions, burns, ulcers, and surgical incisions [27]. In addition, after loading with medications, the sprayable hydrogel can precisely deliver them to the targeted site within the wound area. This localized drug delivery system enhances therapeutic efficacy while minimizing potential systemic side effects [28].

## 3. Design of Sprayable Hydrogel Dressings

**Figure 2:**
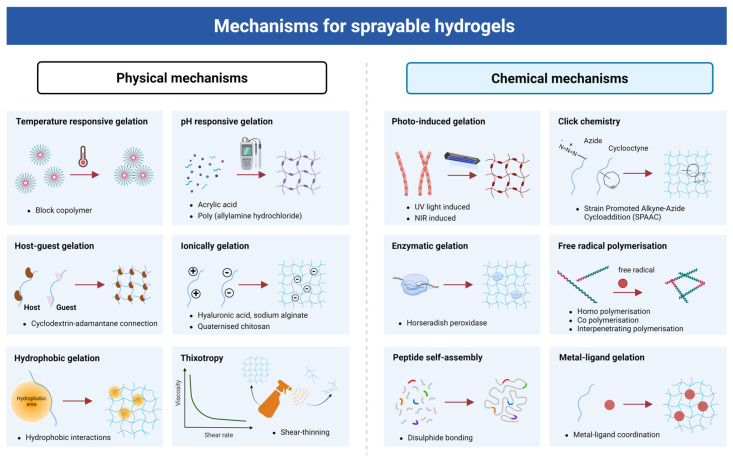
The mechanisms for designing sprayable hydrogels.

The principle of sprayable hydrogels could be mainly based on their unique material properties and preparation technology. When preparing sprayable hydrogels, the polymer is usually dissolved in an appropriate solvent to form a solution suitable for spraying. The solution can be evenly coated on the target surface using spraying technology, such as airbrushes and inkjet printing. These two methods differ significantly in terms of droplet size, shear conditions and formulation requirements. The airbrushes produce larger droplets (50–500 μm), with a lower shear rate (10^3^–10^4^ s^−1^), and are suitable for a wide viscosity range (1–500 mPa·s), making them suitable for large-scale rapid coverage, while inkjet printing generates smaller droplets (10–50 μm), but with an extremely high shear rate (>10^5^ s^−1^), and is only suitable for solutions with low viscosity (<20 mPa·s) and controlled surface tension, making it more suitable for small-area precise spraying. Therefore, in practical applications, the appropriate spraying method should be selected based on the characteristics of the wound and the properties of the formulation. Once the solution touches the target surface, the polymer is quickly crosslinked to form a hydrogel layer through physical or chemical interaction (Figure 2). Physical crosslinking primarily involves temperature responsiveness, pH responsiveness, host–guest interactions, ionic complexation, hydrophobic associations, and shear-thinning effects. Most of these methods operate under mild conditions and exhibit excellent biocompatibility, enabling hydrogels to rapidly form, self-repair, adhere to tissues, and resist loss. They can conform to irregular wounds while maintaining a moist healing environment. Chemical crosslinking methods include photoinitiation, click chemistry, enzymatic reactions, free radical polymerization, peptide self-assembly, and metal coordination. These techniques create stable, mechanically tunable covalent networks. They offer precise control over gelation, facilitate active molecule loading, enhance cell affinity, and promote tissue regeneration. The choice of gelation mechanism directly influences sprayability, adhesion, mechanical strength, and biological functions of hydrogels, thereby significantly affecting their performance in critical wound-healing processes such as hemostasis, anti-inflammation, cell adhesion and proliferation, and tissue repair. The principle of sprayable hydrogels also involves their smart, responsive properties, such as by incorporating components with specific responsiveness (e.g., temperature, pH, ionic, etc.) into the hydrogels [22,29,30]. For instance, in temperature-responsive hydrogels, the swelling degree changes with ambient temperature, which affects their moisturizing and drug release performance, among other properties. Specifically, the temperature change will induce the transformation between gel and sol phases [31]. In essence, the concept of sprayable hydrogel relies primarily on its distinctive material properties and preparation techniques. Polymer material is applied to the target surface using spray technology to create a hydrogel layer with capabilities such as moisturization and drug delivery. The materials used to produce sprayable hydrogels primarily include natural and synthetic polymers, as well as nanocomposites.

## 4. Materials for Preparing Sprayable Hydrogel Dressings

### 4.1. Natural Polymers

The molecular chains of natural polymers have a large number of reaction sites, providing ample space for the design and construction of hydrogel networks. Due to the similarity in chemical composition and biological systems, hydrogels composed of natural polymers usually exhibit good biocompatibility with the human body [32,33]. Moreover, most natural polymers can degrade in the body or disappear in the ecological environment, with good natural abundance and sustainability [17,34]. There are two common types of natural hydrogels, including polysaccharides and proteins. Polysaccharides include chitosan, hyaluronic acid, alginate, cellulose, agarose, glucan, chondroitin sulfate, etc. Proteins include gelatin, fibroin protein, collagen, etc. (Table 2) [11,35].

#### 4.1.1. Chitosan

Chitosan (CS) is a linear polysaccharide consisting of (β-1–4) N-acetyl-D-glucosamine units. Chitosan can be obtained from the shell chitin of crustaceans, such as shrimp, crabs, etc., through deacetylation [14]. Chitosan has excellent biosafety, degradability, antioxidant properties, and low toxicity, making it highly promising for biomedical tissue engineering [36]. The molecular chain of chitosan contains numerous active groups, such as amino and hydroxyl groups [37]. It is the only natural polymer with a positive charge, exhibiting remarkable antibacterial activity against both Gram-positive and Gram-negative bacteria [38]. Chitosan also possesses the unique ability to form complexes with various substances, including metal ions, polymers, lipids, proteins, and DNA [39,40]. Despite its numerous advantages, chitosan still has several limitations, including poor water solubility, rapid in vivo degradation, a need for improved blood compatibility, and relatively weak mechanical properties. To address these challenges, it is often necessary to combine chitosan with other functional materials to broaden its applications and enhance its performance [41,42,43].

Chemical modification approach is very important in broadening its biomedical applications, including alkylation, carboxylation, quaternization, and graft copolymerization [44]. For example, a Janus wettability hydrogel functionalized bandage with a porous gradient wettability channel could be developed based on chemical modification in chitosan for unidirectional drug delivery and wound care [45]. The hydrogel could be fabricated using chitosan quaternary ammonium salt (HACC), poly(vinyl alcohol) (PVA), and poly(acrylic acid) (PAA), and hydrogel exhibited good biocompatibility, excellent antibacterial properties, rapid hemostatic ability, and promote wound healing. Huang et al. introduced a sprayable hydrogel based on chitosan with incorporated silver nanoparticles, exhibiting strong antibacterial properties [46].

**Table 2:** Natural polymers used for fabricating sprayable hydrogel dressings.

Natural Polymer	Chemical Structure	Hydrogel Form	Types of Studies In Vitro/In Vivo	Main Properties	Ref.
Chitosan	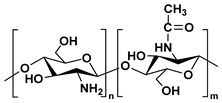	① Sulfonic acid betaine and p-coumaric acid functional groups were introduced onto the main chain of chitosan and cross-linked by glutaraldehyde. ② Gels are formed with lignin through non-covalent interactions.	① Cytotoxicity study and live/dead cell staining, in vitro antibacterial assays, and in vitro cell migration assay. ② Drug release behavior.	Antioxidant and anti-ROS properties, antibacterial performance, promotion of cell proliferation and migration, anti-inflammatory effects and biocompatibility. ② Anti-ultraviolet, anti-oxidation.	[30,47,48]
Hyaluronic acid	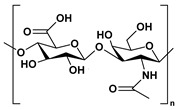	Dopamine and phenylboronic acid modified photocurable hyaluronic acid form sprayable Janus hydrogels through borate ester bonds and photopolymerization.	In vitro fistula model, in vitro anastomosis model, in vitro pressure test model, cell compatibility of GES-1 cells, blood compatibility of rat red blood cells, cell adhesion experiment, repair of rat gastrointestinal perforation, and in vivo biocompatibility of rats.	Promote cell proliferation and migration, anti-inflammatory, anti-adhesion after surgery, and self-healing.	[49,50]
Alginate	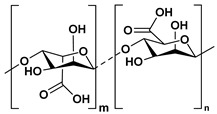	① Cross-linked with chitosan quaternary ammonium salt through Ca^2+^. ② Cross-linked with sodium carboxymethyl cellulose through Ca^2+^.	① Diabetic mouse wound model. ② Cytotoxicity test, cell proliferation test, antibacterial activity test, and rat dorsal perforation wound model.	① Fast cross-linking, capable of filling irregular wounds, antibacterial and antioxidant properties, promoting collagen deposition. ② Biocompatibility and cell proliferation activity, antibacterial performance, hemostasis and anti-inflammatory properties.	[13,51,52]
Cellulose	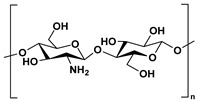	A three-dimensional network structure is formed through electrostatic interactions and hydrogen bonding.	Hemolysis test, CCK-8 test, in vitro coagulation test, SD rat liver hemorrhage model, in SD rat full-thickness skin defect model, histopathological analysis, and porcine full-thickness skin defect model.	Quickly stop bleeding, promote wound healing, anti-inflammatory effect, stimulate angiogenesis, and reduce scar formation.	[53,54]
Agarose	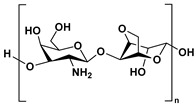	Dopamine is grafted onto the carboxylated agarose.	Cell adhesion and proliferation experiments, drug release experiments, inflammatory response experiments, and adhesion and repair of skin wounds in Balb/c mice.	Promote cell adhesion and proliferation, anti-inflammatory effect, promote wound closure and tissue regeneration, and enhance wound adhesiveness.	[55,56]
Glucan	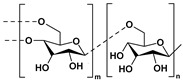	Physical cross-linking with sodium carboxymethyl cellulose.	MTT colorimetric cell proliferation assay and full-thickness excisional wound model of diabetes db/db mice.	Accelerate wound closure, promote the formation of granulation tissue, increase the degree of re-epithelialization, and improve collagen deposition.	[57,58]
Chondroitin sulfate	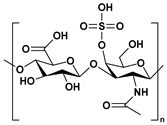	Combining with dopamine through Schiff base reaction and enzymatic cross-linking.	Antibacterial experiments, blood compatibility tests, L929 cell experiments, in vitro degradation experiments, tail transection hemostasis models, liver hemostasis models, and full-thickness skin defect models in rats.	Good biocompatibility, strong tissue adhesion, antibacterial properties, rapid hemostasis ability, and promotion of wound repair.	[59,60]
Gelatin	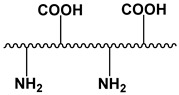	Garlic peroxidase catalyzes cross-linking.	Type I diabetes mouse model, wound tissue histological analysis, and immunofluorescence analysis.	Promote cell migration and tissue repair, accelerate wound closure, enhance the formation of new blood vessels, and promote collagen deposition.	[61,62]
Silk fibroin	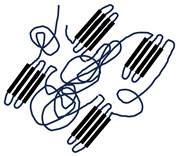	The Schiff base reaction with dopamine and the self-polymerization reaction.	Cytotoxicity test by MTT assay, culture of primary hippocampal neuron, immunofluorescence staining, Western blot, and the adult SD rat model of the T9-10 lateral hemisection spinal cord injury.	Low cytotoxicity, promoting cell adhesion and proliferation, facilitating repair and functional recovery after nerve injury, reducing glial cell proliferation, inhibiting scar formation, and promoting spinal cord injury repair.	[63,64,65]
Collagen protein	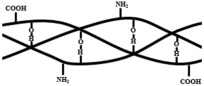	Collagen-binding peptide supramolecular self-assembly.	Ex vivo organ adhesion, the compatibility of 3T3 fibroblasts, and drug-sustained release.	Good tissue adhesion, excellent biocompatibility, and controllable drug release.	[66,67]

#### 4.1.2. Hyaluronic Acid

Hyaluronic acid (HA) is a kind of natural polysaccharide that serves as a primary component of the human interstitium, vitreous body of the eye, synovial fluid of joints, and other tissues [68,69]. Hyaluronic acid plays an important physiological role in water retention, lubrication, and cell repair in the body. Hyaluronic acid exhibits excellent biocompatibility and biodegradability [70,71]. The molecular structure of hyaluronic acid contains numerous hydroxyl and carboxyl groups, resulting in high hydrophilicity. In wound-healing applications, hyaluronic acid can efficiently absorb wound exudates, reduce infection risk, and promote wound healing [72,73]. Due to its large molecular size, remarkable hygroscopicity, and viscoelasticity, hyaluronic acid can regulate tissue hydration and maintain osmotic equilibrium. Hyaluronic acid also contributes to angiogenesis, cell migration, inhibition of inflammation, and stimulation of cytokine activity [74]. In addition to its direct application, hyaluronic acid is often functionalized and chemically modified, and it can be processed and crosslinked to generate multifunctional hydrogels [11]. These properties and biological functions make hyaluronic acid an appealing material for developing biomedical hydrogels for skin wound healing. A sprayable hydrogel wound dressing could be facilely fabricated via introducing methacrylate groups to hyaluronic acid and gelatin [26]. A stable hydrogel could be formed via the controlled photo-crosslinking on the wound surface. The results showed that the obtained sprayable hydrogel could effectively defend against pathogens, provide oxygen at the wound site, relieve metabolic stress in fibroblasts, and reduce cell death under hypoxic conditions in vitro. In addition, this sprayable hydrogel dressing was very beneficial for wound healing due to its practicality, ease of application, and auto-oxygenation and antibacterial properties.

#### 4.1.3. Alginate

Alginate is an anionic polysaccharide, composed of *α*-l-guluronic acid (G) and the *α*-d-mannuronic acid (M) [75]. It is primarily found in the cell walls and intercellular mucilage of brown algae, as well as in certain bacteria, such as Pseudomonas and Azotobacter, which produce mucinous capsules [76]. Alginate reacts with bivalent cations, especially Ca^2+^, to form a gel matrix [77]. In addition, alginate offers good biocompatibility, low cost, and mild gel-forming conditions, making it widely applicable in the biomedical field. Among alginates, sodium alginate is the most commonly used [78]. Several studies have shown that sodium alginate possesses antibacterial and antiviral properties [79] and can be used for drug-sustained release [80], wound treatment [81], and as a hemostatic dressing [82]. Zhang et al. have developed a sprayable alginate hydrogel dressing that produced oxygen and loaded exosomes (EXOs), which could be used to treat diabetic wounds [80]. This sprayable hydrogel contained poly (L-lactic acid) microspheres loaded with calcium peroxide, as well as catalase and exosomes produced by bone marrow mesenchymal stem cells. The obtained sprayable hydrogel could promote local oxygen generation, accelerate macrophage polarization towards M2, and enhance cell proliferation in diabetic wounds. The sprayable composite hydrogel dressing significantly accelerated rapid re-epithelialization, favorable collagen deposition, abundant angiogenesis, thus promoting wound healing.

#### 4.1.4. Cellulose

Cellulose is the most abundant natural polymer on earth and is mainly stored in plants and microorganisms. The molecular skeleton of cellulose is a linear, rigid chain connected by β-D-glucopyranose in a chair conformation via 1, 4-glucoside bonds. The polyhydroxyl groups in cellulose indicate that the cellulose has a high cohesive microfiber network structure [81,83]. The hydrogen bonds make cellulose difficult to dissolve in common solvents [84]. Now, some favorable solvent systems have been explored for cellulose dissolution, such as ionic liquids, deep eutectic solvents, alkali/urea and NaOH/thiourea system, LiCl/N and N-dimethylacetamide (LiCl/DMAc) solvent system, N-methylmorpholine-N-oxide (NMMO), copper-ammonia solution, molten salt hydrates, and quaternary ammonium hydroxides [85,86,87].

In addition to suitable solvent systems, the synthesis of derivatives using the chemical reactions is also considered an effective approach to broaden the applications of cellulose. More commonly used cellulose derivatives are hydroxypropyl methylcellulose (HPMC), hydroxyethyl cellulose (HEC), carboxymethyl cellulose (CMC), and hydroxypropyl cellulose (HPC) [83]. Pan Qi et al. have attempted to deliver in situ cross-linkable double-layer cellulose-based hydrogels via spray [88]. The surface layer of this hydrogel was composed of alginate and carboxymethyl cellulose, which acted as a barrier between the wounded tissue and the surrounding tissue. The bottom layer was composed of alginate and gelatin and came in direct contact with the wounded tissue to promote wound healing. By using appropriate spray conditions, such as a high flow rate and a long spray distance, homogeneous, seamless, double-layered hydrogels could be obtained.

#### 4.1.5. Agarose

Agarose (AG) is a naturally occurring algal polysaccharide that is a renewable and biocompatible biopolymer. Agarose is extracted from a marine red algae and consists of linear chains of agarose, namely alpha-(1,4) and beta-(1,3) glucoside-linked D-galactose and 3,6-anhydro-L-galactose alternating units [89]. The structural units of agarose contain many hydroxyl groups, which easily form hydrogen bonds with hydrogen atoms in the structure or with those of water [90]. Agarose exhibits controlled self-gelling properties, and the resulting agarose hydrogels have adjustable mechanical properties and tunable water adsorption capacity, with excellent potential for various tissue engineering applications [91]. Agarose, as a raw material, has excellent biocompatibility, antibacterial properties, and can promote wound healing, making it highly attractive. However, its structure is simple, and the resulting hydrogel is brittle and contractile, so it can be modified using biological, physical, and chemical methods to improve performance in applications [55]. A research group has used 2,2,6,6-tetramethylpiperidine-1-oxyl (TEMPO) oxidation to degrade and modify agarose, prepared degraded carboxylated agarose to improve the biodegradability, and developed a microgel structure that was easy to spray. In addition, dopamine was added to enhance adhesion. The sustained-release properties, cell proliferation, wound adhesion, and histological changes in agarose-modified hydrogels indicated that the agarose-derived hydrogels promoted the downregulation of proinflammatory cytokines, which were more conducive to wound healing, and had a promising application in skin regeneration and repair [56].

#### 4.1.6. Glucan

Glucan is an isotypic polysaccharide composed of glucose as a monosaccharide, and its glucose units are connected by glucoside bonds. It can be categorized into alpha-glucan and beta-glucan based on the types of glucoside bonds. The main chain of the dextran molecule is linked by β-1,4-glucoside bonds, which are the primary linkages. The side chain is connected to the main chain through β-1,3- or β-1,6-glucoside bonds, forming a dendritic or branched structure [92,93,94]. Dextran is widely found in microorganisms, plants, and animals, including wheat, barley, oats, yeast, bacteria, and fungi. Dextran exhibits good biocompatibility, anti-protein absorption, and is degradable with no toxic side effects. It also possesses antimicrobial properties and is widely used in wound dressings, tissue engineering, and drug delivery systems. Additionally, each glucose unit in dextran has three hydroxyl groups, making it easily modifiable and cross-linkable. By introducing stimulus-responsive groups, functional dextran hydrogels can be developed. Brevé et al. synthesized a photosensitive dextran hydrogel using benzoyl nitride as a crosslinking agent and alkyne-modified dextran by hyperconjugation reaction [95]. Functional dextran-based hydrogels not only maintained hydrophilicity, water retention, and a classic three-dimensional network structure but also exhibited environmental response characteristics, such as temperature and pH responses [95], light responses [96], and so on. Jostein Grip et al. developed a sprayable hydrogel dressing with beta-glucan as the active ingredient for the treatment of both chronic and burn wounds [97]. Carbopol was added to the dressing as a thickening agent to prepare hydrogels of different proportions. All hydrogels were stable by rheological characterization and fluid affinity testing.

#### 4.1.7. Chondroitin Sulfate

Chondroitin sulfate (CHS) is a typical sulfated glycosaminoglycan (GAG) consisting of ~40−100 repeat units of alternating β-1,3-linked-N-acetyl-galactosamine and β-1,4-linked-glucuronic acid sugar residues [98]. CHS is commonly found in animal cartilage and other connective tissues, including blood vessels, ligaments, skin, and tendons, as well as at axon terminals around neuronal cell bodies, in the brain, and in cells surrounding the extracellular matrix [99]. According to the difference in the kinds of uronic acid in its molecular structure and the position of sulfate on hexosamine, it is mainly divided into chondroitin sulfate A (CHS-A), chondroitin sulfate C (CHS-C), chondroitin sulfate D (CHS-D), chondroitin sulfate E (CHS-E), and so on [100]. CHS-based hydrogels could mimic chondrocyte lacunae and create an appropriate environment for cartilage regeneration and function [101]. CHS could promote cell differentiation and provide mechanical protection through tight cell–ECM interactions [102]. In addition to promoting cartilage regeneration [103] and treating knee osteoarthritis [104], CHS also exhibits anti-inflammatory, antioxidant [105], antibacterial [106], anti-tumor [107], immunomodulatory [108], and other pharmacological activities. Wu et al. had fabricated the CHS-based hydrogels by oxidization and dopamine graft of chondroitin sulfate and compositing with carboxymethyl chitosan via the enzymatic crosslinking, Schiff base, and hydrogen bonding interactions [60]. Double cross-linking effectively improved the biological efficacy of the fabricated CHS-based hydrogel and enhanced its antibacterial activity and hemostatic properties. In addition, the hydrogels also demonstrated good cell compatibility, strong adhesion, and spray film formation ability.

#### 4.1.8. Gelatin

Gelatin is a kind of natural polymer material derived from collagen found in animal skin, bones, muscle membranes, and other connective tissues through the process of hydrolysis using acid, alkali, or enzymes [109,110]. Gelatin appears as white or light-yellow translucent, glossy flakes or powder particles. Gelatin exhibits a unique thermos-reversible phase transition behavior due to its chain structure transitioning between random coils and triple helices, allowing it to undergo the gel–sol transition at body temperature. Gelatin has good hydrophilicity, biocompatibility, and degradability. Therefore, the gelatin hydrogel system can be utilized as a drug delivery carrier to load drug molecules for controlled release, and it has been extensively employed in the biomedical field [111]. However, gelatin has poor mechanical properties and instability in physiological environments, which often require modification. For example, gelatin is mixed with natural high molecular compounds (such as cellulose derivatives [112], sodium alginate [113], fibroin protein [114], and hyaluronic acid [115]) and synthetic high molecular compounds (such as polyvinyl alcohol [116], silica [117], and polyethylpyrrolidone [118]) to form the complex that alters the original chemical composition and structure of gelatin. This process not only enhances the original properties of gelatin but also imparts new properties to it. Another common method involves using the functional groups on the gelatin molecular chain to react with low- or high-molecular-weight compounds, thereby altering the properties of the gelatin. For instance, modifying the side groups of the gelatin molecular chain through processes such as acylation, esterification, and other reactions can alter its properties. The aldehydes cross-linked the molecular chains of gelatin to form a new three-dimensional network structure, enhancing the stability, mechanical strength, and thermal properties of gelatin [119]. Liu et al. had created a new type of hydrogel for diabetic wound healing using methacrylic anhydride-modified gelatin hydrogel to mimic neutrophil nanoparticles [120]. This obtained hydrogel exhibited excellent biocompatibility and promoted the growth and proliferation of fibroblasts.

#### 4.1.9. Silk Fibroin

Silk fibroin (SF) is a natural protein derived from silk, with a variety of properties and a wide range of applications. SF is synthesized and secreted by the posterior silk gland cells of the silkworm and processed by the central silk gland and the anterior silk gland, finally forming cocoon silk. In this process, fibroin is encapsulated in the silk fiber along with sericin, but it is usually necessary to remove the sericin by degumming treatment to obtain pure fibroin [121,122]. Silk fibroin is mainly composed of heavy chains (H-chains), light chains (L-chains), and the glycoprotein P25, which are linked by chemical bonds, such as disulfide bonds. The heavy chain is the main component of the silk fibroin protein and contains abundant amino acids such as glycine, alanine, and serine. The arrangement and combination of these amino acids confer unique physical and chemical properties to silk fibroin protein [123,124]. Fibroin protein possesses high strength and toughness, exhibits good affinity for human skin, and is biodegradable. Consequently, silk fibroin has a wide range of applications in the biomedical field, including the production of particles and nanoparticles as drug-delivery carriers [125], the creation of biodegradable scaffolds and films [126], the development of biosensors [127], and the promotion of wound healing [128]. Li Ming et al. had developed an injectable silk fibroin/polydopamine composite hydrogel [64]. The research involved the reaction of the amino group of silk fibroin with the Schiff base formed from the oxidized dopamine quinone. The hydrogel exhibited favorable mechanical and injectable properties.

#### 4.1.10. Collagen

Collagen is a biological polymer that serves as the primary component of animal connective tissues. It is the most abundant and widely distributed functional protein in the human body [129]. It is also one of the most abundant protein components in the extracellular matrix [130,131]. Collagen is mainly divided into many types based on its structure and function. The common types include type I, type II, and type III. Among them, collagen I is the dominant protein, accounting for nearly 70% of collagen in the skin [132]. Collagen I exists in the skin, bone, tendon, blood vessel walls, etc., and is the main component of human connective tissue [133]. Collagen II is mainly found in cartilage and plays a crucial role in the elasticity and flexibility of joints [133]. Collagen III and collagen I work together to form a network that provides tissue support [133]. Collagen forms a fibrous structure that provides elasticity and strength to skin, bone, and other connective tissues [134]. Collagen contributes to the formation of the extracellular matrix and is essential for wound healing and tissue repair [135]. Collagen is biocompatible, biodegradable, and helps stop bleeding [134]; it has become an important biological material in the medical field, used in applications such as sutures [135], tissue repair materials [136], and drug carriers [136]. Shuang Jia et al. have proposed a genetically encoded method to generate protein hydrogels without the need for chemical additives [137]. The desired effect was achieved by genetic encoding of paired cysteine residues at the C- and N-termini of the collagen-like recombination protein. The protein-based hydrogel undergoes a gel–sol transition in response to redox stimulation, achieving a gel–sol transition. The experiment showed that hydrogel improved cell viability, promoted cell migration, and demonstrated good biocompatibility. The cysteine in the collagen-based hydrogel confers it with inherent antioxidant activity, which can reduce oxidative stress and promote healing of diabetic wounds.

### 4.2. Synthetic Polymers

Hydrogels prepared from synthetic polymers have the advantages of high water absorption and retention, softness, shape retention, and biocompatibility [138,139]. The raw materials for synthetic polymer hydrogels mainly consist of alcohol, acrylic acid, and its derivatives (such as polyacrylic acid, polymethacrylic acid, polyacrylamide, polyn-polyacrylamide, etc.) (Table 3) [140]. Their three-dimensional network structure is formed through chemical or physical cross-linking [141]. In the biomedical field, synthetic polymer hydrogels are widely used, particularly in applications that require specific physical properties.

#### 4.2.1. Poly (Ethylene Glycol)

Poly (ethylene glycol) (PEG) is a type of high molecular polymer, and its physical state varies depending on the molecular weight. At room temperature, PEG with molecular weights ranging from 200 Da to 600 Da typically exists as colorless, odorless, viscous liquids, while molecular weights exceeding 600 Da gradually transform into waxy solids or powdery liquids [148]. PEG has good water solubility, dissolves well with many organic components, and exhibits excellent lubricity, moisture retention, dispersion, and adhesion. PEG has excellent biocompatibility, which can help reduce the body’s rejection of the material and the risk of infection. PEG also has non-toxicity, non-immunogenicity, and anti-protein adsorption properties, and is widely used in the biomedical field [149,150]. PEG has an active hydroxyl terminal and can chemically function with a variety of targeting groups or responsive fragments (such as ortho esters, imines, ketals, and acetals). This property allows PEG to be combined with various active drug molecules to create drug delivery systems [151]. For example, the drug molecules could be modified using PEG nanoparticles for enhancing their bioavailability and stability while reducing their toxicity and side effects [152]. PEG is also often designed to be applied as a hydrogel, and many gelation methods (physical, ionic, and covalent interactions) have been reported to form PEG gels. However, covalent crosslinking can lead to the formation of relatively stable hydrogel networks [153]. PEG hydrogels can be used for the delivery of therapeutic agents, tissue engineering, cell culture, 3D bioprinting, bone regeneration, wound healing, and wearable biosensors [154]. Daristotle et al. reported sprayable and intrinsically adhesive wound dressing loaded with antimicrobial silver. The sprayable hydrogel dressing was composed of adhesive and biodegradable poly (lactic-*co*-glycolic acid) and poly (ethylene glycol) blend fibers. This wound dressing was easy to apply, exhibited long-lasting antibacterial properties, was non-cytotoxic, and promoted wound healing [143]. 

#### 4.2.2. Polyvinyl Alcohol

Polyvinyl alcohol (PVA) is a water-soluble polymer made from vinyl acetate through polymerization and alcoholysis. PVA has the characteristics of low price, good solvent resistance, film-forming ability, low protein adsorption, biocompatibility, and biodegradability [155,156]. PVA-based hydrogels are colloidal dispersions with a three-dimensional network structure formed through crosslinking and swelling. They exhibit excellent lubrication properties, low toxicity, high water absorption, good mechanical properties (such as high elastic modulus and mechanical strength), and good biocompatibility [157,158]. The preparation of PVA hydrogels can be divided into two main categories, including physical crosslinking and chemical crosslinking [159,160]. Physical crosslinking is based on weak interactions between or within polymer chains, such as hydrogen bonds and van der Waals forces. The most common preparation method is repeated freeze–thaw cycles, during which the hydrogel can be molded into a shape corresponding to the container. Chemical crosslinking is the process of forming covalent bonds between polymer chains by introducing crosslinking agents via chemical reactions, resulting in a three-dimensional network [161,162,163]. Nevertheless, the physical method of crosslinking is more favored because of restrictions on the use of harmful chemicals as crosslinking agents [164]. Yao et al. had reported a sprayable nanodrug-loaded hydrogel comprising polyvinyl alcohol, gelatin, and glycerol [165]. The hydrogel has high water content and excellent biocompatibility. Dexamethasone sodium phosphate was incorporated into the hydrogel, demonstrating good encapsulation and sustained release of nanodrugs, and the hydrogel exhibited anti-inflammatory and anti-ultraviolet B radiation properties.

#### 4.2.3. Poly(N-Isopropylacrylamide)

Poly(N-isopropylacrylamide) (PNIPAm) is prepared through the polymerization of the monomer N-isopropylacrylamide (NIPAM). PNIPAm is a temperature-sensitive polymer material that can be dispersed in an aqueous solution and respond to changes in ambient temperature [166]. The temperature sensitivity of PNIPAm is associated with its structure. The macromolecular chain of PNIPAm has both hydrophilic amido (-CONH-) and hydrophobic isopropyl (-CH(CH_3_)_2_-) [167]. At low temperatures, hydrogen bonds are formed between the acyl-amino groups and water molecules, resulting in a highly structured solvated layer. The polymer is designed to have an extended coil structure. As the temperature rises, hydrogen bonds weaken, leading to the disruption of the solvation layer around the hydrophobic part of the macromolecular chain. Consequently, the polymer transitions from a loose wire group structure to a tight colloidal structure, exhibiting temperature sensitivity [168]. This temperature sensitivity makes PNIPAm hydrogel promising for various applications across multiple fields. PNIPAm hydrogel can serve as a drug carrier, enabling the regulation of drug release rate and location by manipulating the surrounding temperature [169]. It can also serve as a scaffold material for cell culture and be used to produce temperature-sensitive sensors [170,171]. Pehlivaner Kara et al. had prepared in situ forming thermally and chemically gelling hydrogel based on PNIPAm. A spray gun spray process is used to create a smooth, fast, and conformal hydrogel coating on the surface of intestinal tissue in a site-specific and on-demand manner. This holds promise for the safe and reliable delivery of therapeutic drugs to diseased and damaged intestinal tissues [147].

### 4.3. Nanocomposites

With the rapid development of nanotechnology, scientists have begun to explore the possibility of combining nanomaterials with hydrogels to fabricate sprayable hydrogels. Although traditional hydrogels have good water absorption and retention, they are limited in mechanical properties, biocompatibility, and intelligent responsiveness. The incorporation of nanomaterials can significantly enhance these properties of hydrogels, thereby fostering the emergence and development of nanocomposite hydrogels [172,173]. Nanocomposite hydrogels represent a novel class of materials comprising a water-soluble polymer network and nanoparticles linked through physical or chemical crosslinking [174]. These nanoparticles may consist of inorganic materials (such as nano-hydroxyapatite, carbon nanotubes, nano-silica, etc.) or organic materials (such as nanocellulose, nano-carbon materials, etc.), which interact with the polymer network to form a three-dimensional network structure with special properties [175,176,177].

Nanocomposite hydrogels also have good biocompatibility, high water content, and antibacterial activities. By adjusting the type, content, and distribution of nanoparticles, the mechanical properties of nanocomposite hydrogels can be precisely regulated to enhance toughness, pressure resistance, and resilience [177]. In addition, nanocomposite hydrogels have many new properties that are not available in ordinary organic hydrogels, for example, optical properties (such as fluorescence), magnetism, electrical conductivity, catalytic activity, and self-healing [178,179]. By adjusting the formulation and preparation process, while maintaining the above characteristics, nanocomposite hydrogels can achieve sprayability. Nanocomposite hydrogels can be easily coated on the desired substrate using spraying equipment. Tavakoli et al. proposed a visible light-crosslinkable nanocomposite bio-adhesive hydrogel with multifunctional properties [180]. The authors used Kappa-carrageenan methacrylate as the hydrogel matrix and added polydopamine modified ZnO nanoparticles to enhance its mechanical, antibacterial, and cellular properties. The addition of nanoparticles can significantly improve the tensile strength of hydrogels. The obtained nanoparticles hydrogel had remarkable mechanical properties and recovery ability, comparable elasticity to human skin, good adhesion, antibacterial properties and blood coagulation ability, and was patient-friendly. In addition, L-glutamic acid incorporated into the nanocomposite hydrogel network, as demonstrated in in vivo experiments, has been found to stimulate granulation tissue formation, thereby facilitating wound healing. Sun et al. designed a PEG hydrogel coupled with polydopamine nanoparticles, which can be rapidly solidified by amidation reaction after spraying [181]. The added polydopamine nanoparticles could deliver reactive oxygen species (ROS), induce photothermal effects to enhance bactericidal activity, and exhibit antifouling properties. The reduced polydopamine nanoparticles had redox activity and facilitated the formation of ROS. The near-infrared (NIR) irradiation could accelerate ROS release via the photothermal effect of polydopamine nanoparticles. In vitro experiments have demonstrated the synergistic effect of H_2_O_2_ and NIR-photothermal effects on bacteria. Additionally, in vivo anti-infection experiments have validated the efficacy of polyethylene glycol polydopamine.

## 5. Intrinsic Properties of Sprayable Hydrogel Dressings

**Figure 3:**
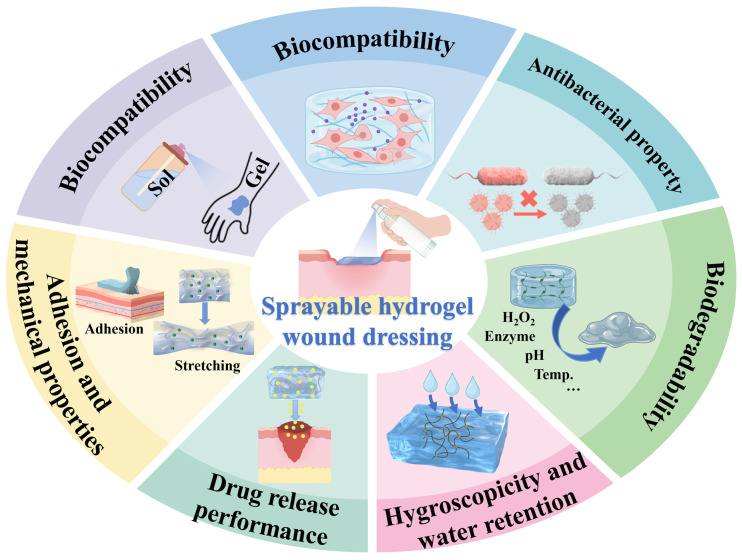
Functions of sprayable hydrogel dressing.

This section describes the physicochemical properties of natural polymers, synthetic polymers, and nanocomposites without reference to specific wound applications. The properties listed here form the basis for the clinical applications discussed in the following section. Sprayable hydrogel dressings with multiple characteristics can facilitate their use in the management of acute and chronic wounds (Figure 3). Next, several desired properties of sprayable hydrogel dressings will be summarized, mainly including rapid spraying, biocompatibility, antibacterial property, biodegradability, adhesion and mechanical properties, drug release performance, hygroscopicity and water retention, and so on.

### 5.1. Rapid Spraying

The core feature of sprayable hydrogel wound dressings is their sprayability, which depends on the design of the delivery system and the rheological properties of the precursor solution [26,182]. Spray delivery systems are primarily categorized into the following two types based on device structure: single-component devices, where the polymer and crosslinker are pre-mixed in the same container and gelation occurs through environmental triggers such as temperature, pH, or UV exposure after spraying, and dual-component spray devices [183], where the polymer solution and crosslinker solution are stored separately and mixed instantly upon spraying, achieving in situ gelation via rapid chemical reactions such as Schiff base reactions or Michael additions [184]. Common spraying techniques include pneumatic spray guns, ultrasonic nebulizers, and handheld aerosol sprayers. The spraying performance of sprayable hydrogels can be evaluated through multiple parameters. For example, the precursor solution can be sprayed onto test paper coated with an indicator (such as CoCl_2_), and the uniformity of coverage can be assessed by color changes or grayscale image analysis. Droplet size distribution can be measured using laser diffraction instruments or high-speed cameras combined with image analysis software. Typically, droplets produced by pneumatic spray guns range from 50 to 500 μm in diameter, while ultrasonic nebulizers generate smaller droplets between 20 and 100 μm. Coverage area can be evaluated by weighing the deposited material or using fluorescent tracing as follows: within a fixed spraying distance and time, the deposition amount per unit area is measured, or a fluorescent dye is added to the precursor solution, and the coverage percentage is calculated after spraying using fluorescence microscopy or image analysis [185]. Based on these design principles and evaluation methods, sprayable hydrogels can be rapidly (within seconds) and uniformly distributed on the wound surface. Whether the wound is flat or irregularly shaped, good adhesion and full coverage are ensured, reducing wound exposure and lowering the risk of infection [181]. Additionally, the cooling effect of sprayable hydrogels, resulting from solvent evaporation, helps relieve pain and discomfort. Their soft texture and strong adhesion to wet tissue reduce friction and irritation, enhancing patient comfort. Compared to traditional dressings, medical staff do not require complex cutting and pasting procedures, which not only saves time but also reduces patient suffering, making sprayable hydrogels particularly valuable in emergency medical care and mass casualty situations. The sprayable hydrogel precursor solution should have shear-thinning properties, with a sudden drop in viscosity at high shear rates to facilitate atomization, and a rapid recovery of viscosity at low shear rates after ejection to maintain the shape of the droplets and promote gelation. The apparent viscosity of the hydrogel precursor solution suitable for spraying is usually within the range of 10–500 mPa·s [186]. The hydrogel formed after spraying should have a storage modulus (G′) higher than the loss modulus (G″), and the G′ value is typically between 100 Pa and 10 kPa to ensure that the gel layer has sufficient mechanical integrity to resist the erosion of wound exudate [187].

### 5.2. Biocompatibility

Sprayable hydrogels used as wound dressings must exhibit excellent biocompatibility to support normal tissue repair [11]. Hydrogels generally demonstrate good biocompatibility because their three-dimensional network structure closely resembles the natural extracellular matrix (ECM) in physical and chemical properties—such as high water content, permeability, and softness—thereby minimizing nonspecific adhesion to proteins and cells [188]. However, biocompatibility is not a binary attribute of “presence or absence is closely influenced by the primary material composition, degradation products, and the concentrations of crosslinking agents, initiators, and other additives”. Currently, biocompatibility evaluation of sprayable hydrogels typically involves in vitro cell viability assays, hemolysis tests, and in vivo animal studies [189]. Each method has limitations as follows: in vitro cell viability assays are efficient but cannot replicate the dynamic wound microenvironment, including inflammatory factors and immune cell infiltration; hemolysis tests assess only the acute toxicity to red blood cells and does not evaluate chronic responses following long-term implantation; and in vivo studies, while more clinically relevant, face challenges such as species differences, ethical concerns, and individual variability. Therefore, an ideal biocompatibility assessment should adopt a multidimensional integrated approach—combining high-throughput in vitro screening, in vitro tissue culture models, and in vivo validation—while emphasizing the long-term safety of material degradation products. Additionally, for sprayable hydrogels, there is currently a lack of systematic research on whether the shear forces during spraying damage biological macromolecules or induce instantaneous toxicity changes in the precursor solution. This represents a critical area for future investigation.

### 5.3. Antibacterial Property

As wound dressings, sprayable hydrogels have strong antibacterial properties, rapidly eliminating bacteria and exhibiting broad-spectrum antibacterial activity. Hydrogel wound dressings create a physical barrier that protects the wound surface, preventing external bacteria from entering [6,190,191]. Simultaneously, the hydrogel maintains a moist environment on the wound’s surface, promoting wound healing and inhibiting the growth of specific bacteria. Sprayable hydrogels can also be loaded with various antibacterial materials to enhance their antibacterial properties [15]. Sprayable hydrogels can also be loaded with various antibacterial agents, such as silver nanoparticles, antibiotics, and antimicrobial peptides, to enhance their antibacterial properties. These antibacterial agents can be evenly distributed in the hydrogel matrix and gradually released onto the wound surface as the hydrogel swells and degrades, thereby exerting an antibacterial effect [192]. Silver nanoparticles are commonly used as antibacterial agents with a wide spectrum of activity. They can disrupt the bacterial cell membrane and DNA, hindering bacterial growth and reproduction. However, concerns exist regarding their biocompatibility and dose-dependence. Moreover, the excessive use of conventional antibiotics has led to the emergence of drug-resistant bacteria. Antimicrobial peptides (AMPs) have the potential to exhibit broad-spectrum antibacterial activity against Gram-positive and Gram-negative bacteria, fungi, and protozoa, demonstrating high efficacy. Combining antimicrobial peptides with hydrogels to create antimicrobial peptide hydrogels can help overcome the aforementioned limitations and present a more promising application outlook [193]. Unlike traditional antibiotics which tend to induce drug resistance through metabolic inhibition, antimicrobial peptides kill bacteria through a physical membrane disruption mechanism and are less likely to develop resistance [194]. The antimicrobial peptide hydrogel forms a physical barrier combined with local controlled release and can efficiently kill bacteria at a lower concentration, avoiding the dose-dependent toxicity of silver nanoparticles; by covalently fixing or physically embedding the antimicrobial peptides in the three-dimensional network of the hydrogel, it can provide physical protection for them, significantly prolonging their action time in the wound microenvironment, and achieving sustainable release [195]. Annabi et al. used antimicrobial peptides to design a sprayable, elastic hydrogel adhesive with antibacterial properties and biocompatibility. This adhesive can be employed in sutured wound closure techniques to prevent infections and promote healing of chronic wounds [15].

### 5.4. Biodegradability

Sprayable hydrogel wound dressings need to be biodegradable. By naturally degrading into non-toxic or low-toxic products, they can not only reduce the environmental burden but also create a more suitable microenvironment for cell growth and repair at the wound site, thereby effectively promoting rapid wound-healing and regeneration [27,196]. However, the degradation behaviors of different materials vary significantly, and the ideal degradation time should align with the wound healing process. In sprayable hydrogel systems, various natural and synthetic degradable materials have been extensively studied. The guar gum/chitosan hydrogel, cross-linked through a Schiff base reaction, retains approximately 40% of its initial mass after 28 days in PBS. It can accelerate tissue repair by about 99% within 14 days, making it suitable for chronic wounds that require several weeks to heal [197]. The PVA-hyaluronic acid/ZnO-AV sprayable hydrogel (with a ZnO/AV ratio of 3:1) exhibits a mass loss of 41% within 14 days, significantly lower than the 60% mass loss of pure PVA-hyaluronic acid. This indicates that the introduction of ZnO enhances mechanical properties while delaying the degradation rate, making it more suitable for wound-healing processes requiring stable support [145]. Metal ion regulation enables the hydrogel to self-heal within only 7–8 s after injection and achieve complete degradation within 37 h, making it suitable for acute wounds or hemostasis scenarios that require short-term temporary coverage [198]. The carboxymethyl chitosan/polyphenol sprayable hydrogel achieves a wound-healing rate of 93.98 ± 0.63% on the 10th day in a full-thickness skin defect rat model, and its degradation rate is well-matched with the tissue regeneration process [199]. In general, for a surface wound dressing, optimal degradation occurs within a week [200]. If the dressing is loaded with a drug, it should degrade in 21 days so that the drug can fully exert its effects without imposing excessive burden on the tissue [201]. Crosslinking density, material composition, and environmental conditions are important factors in regulating the degradation rate: the higher the crosslinking density, the denser the hydrogel network, and the slower the degradation. Therefore, in practical applications, customized designs should be made based on the type of wound and treatment requirements, choosing materials with faster degradation for acute wounds and slower degradation systems for chronic wounds to achieve the best therapeutic effect.

### 5.5. Adhesion and Mechanical Properties

Sprayable hydrogel wound dressings quickly cure on the wound surface when sprayed, forming a stable gel layer. With strong adhesion, they can effectively conform to irregular wound surfaces, ensuring stable adhesion in frequently moving parts such as joints. Hu et al. developed a sprayable zwitterion antibacterial hydrogel that achieved strong adhesion to the wound surface through the strong electrostatic effect of the zwitterion and modified silver nanoparticles and has great potential to promote joint skin wound healing [25]. The hydrogel exhibits a strong adhesive strength (15–38 kPa). This interfacial adhesion is mainly attributed to the synergistic effect resulting from hydrogen bonds, electrostatic attractions, dipole–dipole interactions, and metal coordination between the zwitterionic portion and the reactive groups on the surface of the attached substance, as well as the adhesion provided by the catechol groups in the hydrogel. However, in the design and application of wound dressings, strong adhesion on both sides is neither necessary nor desirable and may even have adverse effects. If the inner side of the dressing shows excessive adhesion to the normal tissue interface, it may lead to unnecessary tissue adhesion, thereby increasing the risk of infection and hindering the natural healing process of the wound. Similarly, if the adhesion of the outer surface of the dressing is too strong, it is easy to absorb pollutants such as dust and particles in the environment, which not only introduces potential pollution sources to the wound area but also may hinder the cleaning and protection of the wound, which is not conducive to the maintenance of its healing environment [202]. Therefore, achieving asymmetric adhesion is of great importance. Wang et al. developed a sprayable Janus hydrogel with an asymmetric adhesion effect. The adhesive strength of hydrogels is within the range of 5–15 kPa, and the adhesion sites are mainly provided by the catechol groups in dopamine. This hydrogel not only ensures secure adhesion to complex wounds but also possesses inherent mechanical strength for postoperative anti-adhesion, demonstrating significant potential for repairing complex wound surfaces [50].

Sprayable hydrogel with a soft, flexible appearance offers good shape adaptability, allowing it to fit the contours of the wound closely. This reduces the gap between the dressing and the wound, helping to prevent bacterial invasion and infection. Hydrogel dressings must possess a certain degree of elasticity to accommodate the bending, stretching, and other movements of the wound site. Highly elastic dressing can better adapt to the wound’s deformation, reducing discomfort or injury caused by the dressing being too tight or too loose. Sprayable hydrogels exhibit high resilience, quickly returning to their original shape and size after external force is applied, ensuring the stability and durability of the dressing. Additionally, they need to have good tensile properties to withstand large tensile forces without breaking or losing adhesion, better adapting to the deformation and movement of the wound. Ideal for sprayable hydrogels, whose mechanical properties can be adjusted within a range to mimic the natural extracellular matrix (ECM), they regulate the delivery of biochemical compounds and provide mechanical stimulation to 3D cell structures to promote cell function and tissue development [203].

### 5.6. Drug Release Performance

**Figure 4:**
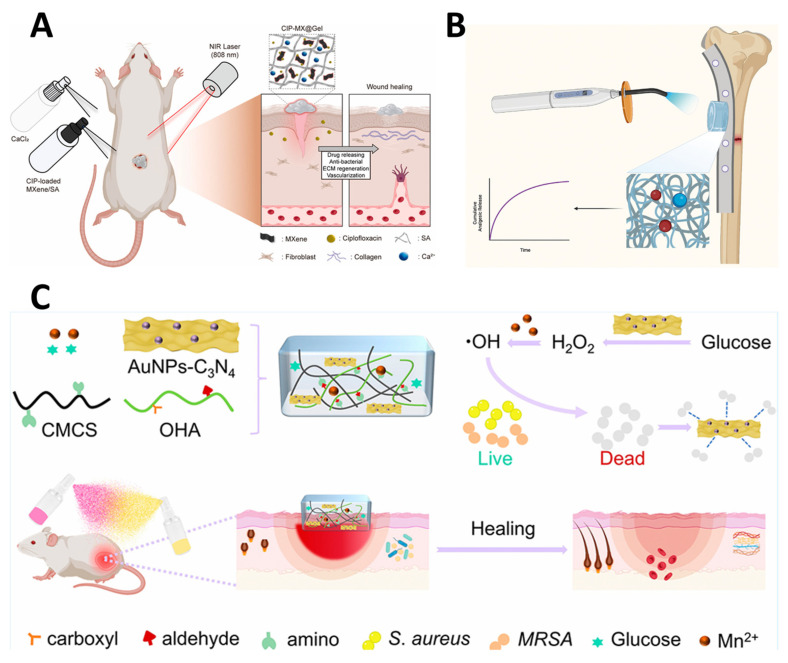
The sprayable hydrogels loaded with drugs could promote the wound dressing process. (**A**) Ciprofloxacin (CIP) could be loaded into an MXene/sodium alginate hydrogel, thereby inhibiting bacterial infections and enhancing wound healing [204]. (**B**) The analgesics and nonsteroidal anti-inflammatory drugs (NSAIDs) could be loaded into methacrylated oligomeric polyethylene gly*co*l-*co*-lactic acid polymer hydrogels for orthopedic applications [205]. (**C**) Au nanoparticle-carbon nitride (AuNPs-C_3_N_4_) nanozyme, glucose, and Mn^2+^ could be incorporated into the sprayable hydrogel fabricated using carboxymethyl chitosan and oxidized hyaluronic acid [206].

The hydrogel is a three-dimensional porous structure that serves as a scaffold for drug loading (Figure 4).

The pore structure is a critical factor in regulating drug release behavior in hydrogels. Generally, larger pores with better internal connectivity offer less resistance to drug diffusion, resulting in a faster release rate. Conversely, smaller pores extend the migration path of drug molecules, reducing the release rate and imparting the hydrogel with excellent sustained and controlled release properties [207]. However, the role of pore size is not isolated; it interacts with the relationship between drug molecule size, the uniformity of the pore network, and the swelling characteristics of the hydrogel [208,209]. Together, these factors determine the drug release kinetics of drug-loaded hydrogels and provide an important theoretical basis for the structural design of targeted and long-acting drug delivery systems. Specifically, drug molecules can be completely released through passive diffusion only when the mesh size of the hydrogel is larger than the hydrodynamic radius of the drug molecule. If the pore size decreases, simple diffusion is hindered, and the release behavior shifts to an “anomalous transport” or “zero-order sustained release” mode, where the dissolution of active molecules depends on the erosion and degradation of the polymer matrix [210]. Loading drugs through hydrogels has many advantages. First, the porous structure of the hydrogel can protect drug molecules from the external environment, thereby improving their stability. Secondly, the porous structure of the hydrogel can protect drug molecules from the external environment, thereby improving their stability. In addition, because the drug is released locally rather than distributed throughout the body, systemic side effects can be significantly reduced. Finally, the slow-release properties of hydrogel enable the drug to remain active in the body for a longer period, thereby improving its bioavailability. It should be noted that not all chemical crosslinking strategies are inherently toxic. For example, crosslinking via four-armed PEG-succinimidyl esters or natural phenolic coupling produces highly biocompatible networks, as demonstrated in FDA-approved tissue adhesives. Li Ming et al. fabricated a ciprofloxacin (CIP)-loaded MXene/sodium alginate (SA) hydrogel that can be sprayed. This hydrogel demonstrates outstanding photothermal conversion and biocompatibility under near-infrared (NIR) irradiation (Figure 4). It also facilitates controlled drug release via NIR irradiation, thereby enhancing antibacterial properties and accelerating wound healing [204]. In addition to their application as wound dressings, sprayable hydrogel-supported drugs can also be utilized in various other medical domains. For instance, chemotherapy drugs are encapsulated within hydrogels and administered directly to tumor tissue through local injection or implantation for cancer treatment. Hydrogels serve as cell scaffolds and are loaded with bioactive substances, such as growth factors, to facilitate tissue repair and regeneration.

### 5.7. Hygroscopicity and Water Retention

The interior of the hydrogel features a unique three-dimensional network with numerous tiny pores that can adsorb and retain a large number of water molecules. Hydrophilic groups in hydrogels (such as -OH, -CONH-, -CONH_2_-, -SO_3_H, etc.) also enhance their water absorption capacity. When water molecules penetrate the hydrogel network, they undergo physical adsorption on the surface of the hydrogel, thus being securely preserved within it. The hydrogel maintains its shape and structure effectively after water absorption, resists dissolution, and exhibits excellent water retention performance. The hygroscopic and water-retaining properties of hydrogel wound dressings are very important for wound healing. They create a moist healing environment that supports the growth and regeneration of wound epithelial cells, promotes collagen synthesis, and accelerates wound healing [211]. Moreover, the moist environment aids in reducing wound pain and discomfort, enhancing patient comfort. By absorbing wound fluid, hydrogel wound dressings reduce the bacterial load on the wound surface, lowering the risk of infection. Wang et al. incorporated hyaluronic acid (HA) into the liquid crystal state liquid crystal (LLC) spray dressing, significantly enhancing the material’s water absorption and mechanical strength. This dressing, with its excellent moisture absorption and retention capabilities, can effectively cover exudative wounds for extended periods, maintain a moist healing environment, improve patient compliance, and show promising potential for clinical applications [212]. In addition, the water absorption and moisture retention properties of hydrogel are closely related to its swelling behavior and affect its rheology, expansion behavior and drug release. High water absorption can promote the full hydration and expansion of polymer molecular chains, reduce the network crosslink density and structural rigidity, and weaken the material elasticity and enhance its flexibility. For example, the G′ of the chitosan/gelatin photo-crosslinked hydrogel can be adjusted within the range of 12.2–291.0 Pa, and a higher G′ often leads to a shift in drug release from the Fickian diffusion mechanism [213]; after adding 0.2 wt% single-walled carbon nanotubes to the guar gum hydrogel, G′ increases from 0.2 MPa to 2.2 MPa, and the drug release changes from a sudden release of 96% within 4 h to zero-order sustained release over 28 h [214]. At the same time, after the hydrogel absorbs water, it expands internally, forming a loose and porous network structure. This can broaden the drug diffusion channels and accelerate the drug release rate. Therefore, hygroscopicity and water retention have a multi-faceted impact on the application effect of sprayable hydrogels.

### 5.8. Self-Healing Ability

Sprayable hydrogels with self-healing ability are emerging as a highly adaptive platform for sealing and protecting dynamic wound sites, where conventional dressings are prone to fracture, delamination, and loss of barrier function. Their autonomous repair capability is typically imparted by reversible crosslinking networks that can dissociate under strain and subsequently re-associate to restore structural integrity. Self-healing sprayable hydrogels could be designed via involving dynamic covalent bonds (e.g., Schiff base, disulfide, or boronate ester linkages) and non-covalent interactions such as hydrogen bonding, host–guest complexation, or metal–ligand coordination [215]. For example, a sprayable chitosan-based hydrogel could be designed using Schiff base bonds between amino groups of hydroxypropyl chitosan/caffeic acid-grafted chitosan and aldehyde groups of oxidized dextran. This dynamic imine network enabled rapid self-healing after high-strain damage, as confirmed by step-strain rheological testing, significantly extending the dressing’s service life under cyclic deformation [216]. The researcher also fabricated a polysaccharide hydrogel combining Schiff base linkages, enzymatic crosslinking, and hydrogen bonding, which exhibited complete mechanical recovery after rupture and maintained strong tissue adhesion during repeated bending and twisting [60]. Our group has also tried to incorporate polydopamine nanoparticles into a methacrylated hyaluronic acid-based sprayable hydrogel. Then, the abundant catechol groups could contribute to reversible hydrogen bonds and π–π stacking, imparting good cyclic compressibility and energy recovery [217]. By autonomously resealing micro-cracks, such self-healing sprayable hydrogels preserve a moist, infection-resistant barrier, reduce the need for frequent dressing changes, and are particularly advantageous for wounds on moving body surfaces.

### 5.9. Antioxidant and Anti-Inflammatory Properties

For managing chronic wounds, such as diabetic ulcers, the sprayable hydrogels should possess antioxidant and anti-inflammatory properties to persistent oxidative stress and dysregulated inflammation critically impair healing. The sprayable hydrogels can be loaded with natural polyphenols (e.g., epigallocatechin gallate, gallic acid) or synthetic antioxidant moieties that scavenge excessive reactive oxygen species (ROS) and modulate inflammatory cascades. The direct radical neutralization could reduce oxidative damage to cellular lipids, proteins, and DNA, while concurrently suppressing pro-inflammatory cytokines and promoting macrophage transition from the pro-inflammatory M1 to the reparative M2 phenotype [218]. Liu et al. have developed an in situ-forming sol-spray hydrogel incorporating itaconic acid and gallic acid into a sodium alginate/PVA network. The fabricated hydrogels exhibited potent DPPH radical scavenging, significantly reduced intracellular ROS in HUVECs, downregulated TNF-α and IL-1β, and upregulated IL-10, thereby accelerating diabetic wound closure in mice [219]. Mao et al. also engineered a self-reinforcing sprayable hydrogel with polydopamine nanoparticles that showed over 80% DPPH scavenging and effectively cleared ROS from L929 cells, while reducing CD86^+^ M1 cells [220]. In addition, a sprayable ROS-responsive hydrogel coating bearing an ethyl caffeine prodrug was also fabricated, disintegrating in high-ROS environments and, upon release, scavenged ABTS/DPPH radicals, restored endothelial tight junctions, and mitigated post-interventional vascular inflammation [221]. The integration of antioxidant/anti-inflammatory functions into sprayable hydrogels could effectively reduce secondary tissue damage, create a pro-regenerative microenvironment, and accelerate wound repair [222].

### 5.10. Hemostatic Performance

For acute wounds, rapid hemostasis is a critical prerequisite to prevent exsanguination and create a pro-regenerative microenvironment. Sprayable hydrogels could also address this need via enabling immediate, conformable application to irregular bleeding surfaces. The rapid absorption of blood concentrates endogenous clotting factors, while cationic polymers electrostatically interact with negatively charged erythrocyte and platelet membranes, activating the coagulation cascade [223]. The in situ-forming porous network serves as a physical barrier and scaffold for clot stabilization. For example, a sprayable oxidized cellulose nanofiber hydrogel was designed with superior clotting efficiency in rat liver and porcine skin defects by rapidly absorbing plasma and forming a tight physical seal [54]. Another self-gelling hydrogel was fabricated via chitosan/ε-polylysine/poly(methacrylic acid) powder, a clotting index of 95.3% was achieved, and the bleeding time was reduced to ~15 s in a rat liver model, attributed to its strong positive charge and fast hydration [224]. Furthermore, a sprayable crosslinker-free Schiff-base hydrogel of oxidized alginate and carbohydrate-modified gelatin, delivered via an air-assisted system, provided rapid, compression-free hemostasis superior to commercial sponges [225].

### 5.11. Oxygen Permeability and Gas Exchange

Sprayable hydrogels designed for wound healing could also balance moisture retention with adequate oxygen permeability, considering that cellular respiration and angiogenesis critically depend on a sustained oxygen supply. Gas exchange is mainly governed by the hydrogel’s pore architecture, thickness, and hydrophilicity [11]. To actively counteract hypoxia, especially in chronic or ischemic wounds, some formulations incorporate oxygen-generating agents such as calcium peroxide (CaO_2_). Upon contact with aqueous media, CaO_2_ decomposes to release hydrogen peroxide, which is subsequently converted by catalase into molecular oxygen, providing sustained local oxygenation. A photocrosslinkable gelatin/hyaluronic acid sprayable hydrogel contained CaO_2_ was fabricated to release oxygen under hypoxic conditions [26]. Wu et al. have engineered a breathable, porous Ecoflex-encapsulated hydrogel oxygen sensor, the 152 µm-thick porous elastomer film selectively permitted O_2_ permeation while blocking liquid water, enabling real-time monitoring of transcutaneous and dissolved oxygen [226]. Parshad et al. also designed a hydrogel nanocomposite (polysulfobetaine/PEG/TiO_2_) with micrometer-scale pores that allowed efficient gas diffusion to an underlying porphyrin sensor, achieving accurate pO_2_ measurements in blood within an extracorporeal circuit [227].

### 5.12. Conformal Coating Ability

Sprayable hydrogels designed for shallow wounds (e.g., abrasions, superficial burns) should form ultra-thin, uniform, and conformal coatings that adhere to micro-topography without compromising breathability or causing maceration. This capability is achieved through low-viscosity precursors that readily spread, coupled with rapid interfacial crosslinking. A powder-based spray system comprising polyacrylic acid/polyethyleneimine microparticles could be designed for mist spraying; the particles coalesce within seconds into a conformal hydrogel skin that faithfully replicates substrate microstructures [228]. He et al. have highlighted the sprayable dressings for burns exploit shear-thinning behavior and rapid evaporation to form adherent films that adapt to irregular geometries [27]. The conformal coatings could also preserve gas exchange and moisture balance while preventing maceration, making them ideal for superficial wound management.

### 5.13. Others

Except for the above properties of the sprayable hydrogels, there are still other properties that could be explored, such as the sprayable hydrogels that could be designed for skin graft fixation, providing immediate tackiness to prevent graft shearing, maintaining a moist interface, and permitting host-to-graft cell migration without cytotoxicity. For example, a catechol-functionalized GelMA-DOPA hydrogel exhibited strong wet adhesion (~20 kPa) and supported fibroblast/keratinocyte viability, enabling seamless graft integration [182]. To retain injectable scaffolds (e.g., microspheres, cells, or growth factors) at the wound site, the hydrogel should possess high yield stress and rapid recovery after spraying. A dual-network microgel adhesive swelled to >900% within seconds, forming a cohesive barrier that prevented washout by exudate while providing sustained drug release for >10 days [229]. Furthermore, sprayable hydrogels can be used as adjuncts to sutures without compromising holding strength. Thermosensitive Pluronic F127-based formulations remain low-viscosity at room temperature for easy spraying, then gel at body temperature, allowing needle penetration without tearing and maintaining suture integrity [230]. These multifunctional sprayable hydrogels serve as effective “bio-glues” for graft fixation, as retention matrices for injectable therapeutics, and as suture-compatible barriers, meeting the demands of complex wound management [231,232].

## 6. Wound-Healing Applications

According to the different wound types, a clinically significant complication should be clearly defined as currently exceeding acceptable levels with standard care (e.g., topical gels, gauze, or ointments). A sprayable hydrogel, namely, the ability to form an ultra-thin, conformal, rapidly setting layer, can reduce that complication to below an acceptable threshold. In contrast, dual-syringe topical gel, while excellent for filling deep cavities or delivering bulk material, cannot achieve the same uniform thin coverage on large, irregular, or moving surfaces without dripping or pooling. Moreover, sprayable hydrogels are particularly suited for partial-thickness burns, skin graft fixation, and retention of injectable scaffolds.

### 6.1. Diabetic Wound Healing

**Figure 5:**
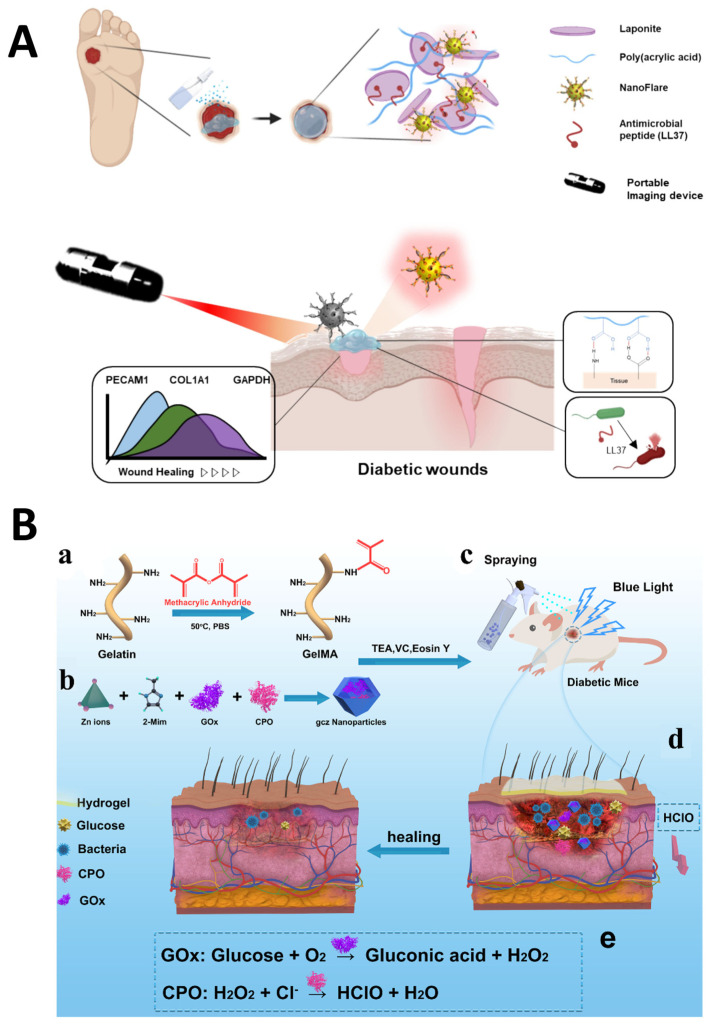
Sprayable hydrogel wound dressings for diabetic wounds. (**A**). Schematic illustration of a sprayable hydrogel (SH) with NanoFlares and antimicrobial peptides (LL37) for monitoring diabetic wounds and promoting wound-healing [236]. (**B**). Schematic diagram of the preparation of GelMA hydrogel with loaded biomimetic neutrophils and its wound healing process in diabetic rats [120].

In recent years, the global prevalence of diabetes has shown an increasing trend, making it one of the major public health problems worldwide. Diabetic patients often experience a more complex and slower wound-healing process due to high blood sugar levels, decreased immune function, poor blood circulation, and other factors. For non-exuding, flat diabetic ulcers, an injectable hydrogel (e.g., dual-syringe alginate) is often sufficient and easier to apply. However, when the ulcer is large, irregular, or located on a moving joint, a sprayable formulation provides more uniform coverage and better adherence—a niche advantage that justifies the additional complexity. Sprayable hydrogel wound dressings offer significant advantages and potential in diabetic wound healing (Figure 5) [233]. Liu et al. created a sprayable hydrogel composed of methacrylic anhydride-modified gelatin (GelMA) that mimics neutrophil nanoparticles. Nanoparticles mimicking neutrophils are encapsulated in ZIF-8 nanoparticles, which consume glucose to produce HClO, thereby decreasing the glucose concentration in the wound and inhibiting bacterial growth. The hydrogel has good biocompatibility, promotes the growth and proliferation of fibroblasts, accelerates wound healing in type I diabetic rats, and has great potential for the treatment of diabetic wounds [120]. Adding intelligent substances to the hydrogel that respond to factors such as temperature, pH, glucose concentration, etc., can enable on-demand drug release and enhance the healing of diabetic wounds. Tricou et al. developed a zeolite-loaded hydrogel for diabetic wound healing. Zeolites were encapsulated in a calcium-crosslinked alginate hydrogel, which regulates pH at the wound site. This hydrogel can effectively neutralize hydroxide ions in serum-containing simulated wound fluid and can be used as a treatment for diabetic foot ulcers (DFUs) [234]. Diabetic wounds may suffer from local hypoxia. Sprayable hydrogels combined with nanoparticles can provide sufficient oxygen supply to the wounds, promoting cell proliferation and collagen synthesis. Combining hydrogels with other materials, such as fibers and microspheres, to create composite dressings can harness the benefits of different materials and improve the effect of diabetic wound treatment. In diabetic foot ulcers, the clinical complication of non-healing due to biofilm formation and hypoxia exceeds an acceptable level (e.g., up to 25% of ulcers result in amputation) [235]. A sprayable hydrogel loaded with oxygen-generating nanoparticles and antimicrobial peptides can be applied as a thin, breathable layer that conforms to the irregular ulcer bed, something a thick topical gel cannot do without causing maceration. The rapid in situ gelation creates a moist but non-occlusive barrier, reducing bacterial load and promoting granulation.

### 6.2. Healing of Infected Wounds

**Figure 6:**
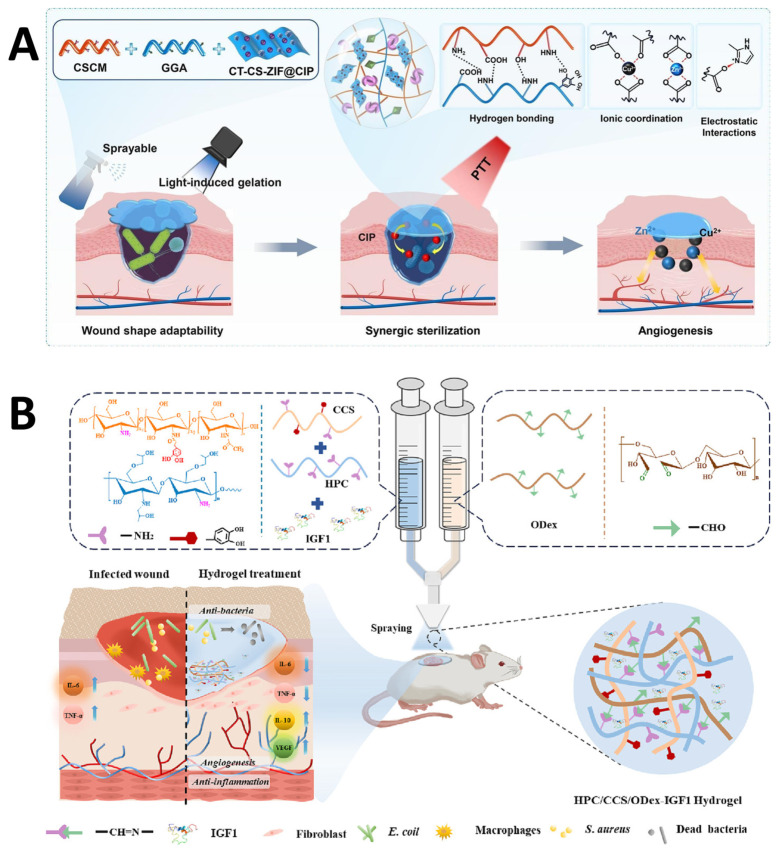
Sprayable hydrogel wound dressings for infected wounds. (**A**) Chitosan- and gelatin-based sprayable hydrogels for the management of infected wounds [240]. (**B**) Sprayable, self-healing chitosan-based hydrogels for promoting the healing of infected wounds [216].

For infected wounds, the risk of sepsis from uncontrolled bacterial proliferation is unacceptably high with conventional dressings. A sprayable hydrogel containing silver nanoparticles or antimicrobial peptides can be applied as a conformal coating that covers every crevice of the wound, whereas a syringe-applied gel may miss small pockets. The thin layer also allows oxygen exchange, inhibiting anaerobic bacteria. The healing of infected wounds after surgery is an important medical issue. It is a complex process influenced by several factors, including the severity of the infection, the wound’s location, and the patient’s overall health. For post-operative infected wounds, hydrogel dressings are anti-infectious, helping control infection spread and reduce bacterial growth. Hydrogels can mimic the natural extracellular matrix, providing an environment conducive to wound healing and promoting the healing process (Figure 6) [237]. Wang et al. developed a nanodiamond hydrogel for the healing of infected wounds and cancer treatment [238]. Nanodiamonds (NDs) have excellent biocompatibility and are added to a solution of acrylic-grafted chitosan (CEC) and oxidized hyaluronic acid (OHA) to create a multifunctional hydrogel. The hydrogel exhibits rapid hemostasis, promotes wound healing, possesses excellent self-healing and injectability, enhances the healing of infected wounds, and inhibits tumor cell proliferation. Sprayable hydrogels can be applied directly to the wound surface, rapidly forming an antibacterial barrier that effectively inhibits and kills bacteria in the wound’s vicinity, thereby reducing the risk of infection. Additionally, there is no need for direct contact with the wound during spraying, which minimizes patient pain and discomfort. Therefore, sprayable hydrogels offer a significant advantage in treating postoperative infected wounds. Xiao et al. designed a lyotropic liquid crystal (LLC)-based bacteria-resistant and self-healing spray dressing loaded with ε-polylysine (PLL). PLL kills resistant bacteria, and the cubic cells of LLC were used to encapsulate PLL to enhance its stability and induce sustained release, thereby achieving a long-term antibacterial effect. Hydrogel dressing exhibits good antibacterial activity, excellent water retention, and outstanding mechanical properties. It significantly contributes to the healing of post-operative infected wounds [239].

### 6.3. Postoperative Adhesion

**Figure 7:**
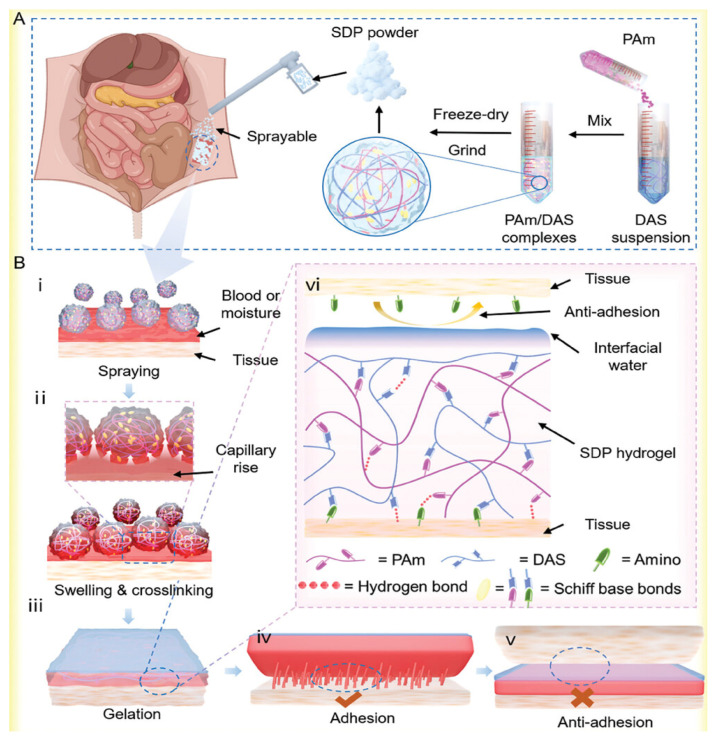
Sprayable hydrogel wound dressings for postoperative adhesion. (**A**) A schematic illustrates the application of sprayable hydrogels to injuries of the cecum and abdominal wall. (**B**) The gelation process of sprayable hydrogels was outlined [244].

Postoperative adhesion is a prevalent clinical condition resulting from surgical trauma, particularly in abdominal, pelvic, pericardial, and tendon surgeries. The development of adhesions is associated with various factors, including surgical trauma, inflammatory response, tissue healing, and fibrosis. Postoperative adhesions can cause severe or chronic pain, intestinal obstruction, and other symptoms, substantially diminishing patients’ quality of life (Figure 7). However, few current treatments are consistently effective in preventing post-operative adhesions. The porous structure of the hydrogel can accommodate various drugs and regulate drug release. Evidence from existing studies has confirmed the feasibility and superiority of using hydrogels to counter postoperative adhesions, primarily due to their outstanding antifouling ability [241,242]. Sprayable hydrogel wound dressings can quickly form a protective film on the wound surface after application, separating the damaged tissue from surrounding tissues/organs. This film acts as a physical barrier, effectively preventing the damaged tissue from coming into direct contact with surrounding tissue and reducing the formation of adhesions. Chen et al. designed a sprayable multifunctional nanoparticle-in-microgel system (nMGel hydrogel microspheres) with rapid hemostatic and antioxidant properties. nMGel can be applied as a powder spray, exhibits good tissue adhesion, and can prevent the formation and progression of postoperative adhesions through its hemostatic, antioxidant, and anti-inflammatory effects. It can conform to any irregular injury site, is user-friendly, highly biocompatible, and efficiently inhibits postoperative tissue adhesion [243]. Zou et al. prepared a degradable spray glycyrrhetinic acid hydrogel (GAG) based on natural glycyrrhetinic acid (GA) by straightforward heating and cooling without the use of any additional chemical cross-linking agents to prevent postoperative adhesion. The GAG has superior anti-inflammatory activity, excellent degradability, and biocompatibility. It also demonstrates outstanding antibacterial activity against *S. aureus* without causing cytotoxicity. GAG can be used as a physical barrier material as well as an additional therapeutic agent to effectively minimize the development of postoperative peritoneal adhesions by reducing the inflammatory response and collagen fiber deposition [17]. In conclusion, sprayable hydrogel wound dressings have significant advantages in preventing postoperative adhesion. Through their physical barrier effect, biocompatibility, hemostatic function, antioxidant and anti-inflammatory effects, and ease of use, hydrogel dressings can effectively reduce the incidence of postoperative adhesions and improve patients’ quality of life.

### 6.4. Tumor Treatment

Cancer is a major health problem and the second leading cause of death worldwide, affecting millions of people each year. Hydrogels play an important role in tumor therapy and can be used as spacer materials for radiation therapy, carriers of anti-tumor drugs, and as an adjuvant tool for immunotherapy [245]. Sun et al. developed an injectable, reactive oxygen species biodegradable therapeutic hydrogel (ADU-AAV-PD1@Gel) for a glioblastoma (GBM) surgical resection model. The hydrogel system includes solute PD-1 (sPD-1), developed with adeno-associated virus type nine as a carrier for gene immunotherapy, and ADU-S100, an agonist of STING, a key protein regulating innate immunity, to achieve continuous infiltration of T cells and recovery of effector function. When injected into the resected tumor cavity, the hydrogel system consistently released sPD-1 and ADU-S100 in response to the large amounts of ROS released by combined radiotherapy. Simultaneously, the natural immune regulatory protein STING was activated and the PD-1/PD-L1 pathway was blocked. This exerted a strong anti-tumor effect on radiotherapy-immune responses and induced a lasting immune memory, effectively inhibiting the recurrence of postoperative GBM [246]. Sprayable hydrogels can be directly applied to the tumor site by spraying, making the process simple and fast. This method reduces the complexity and risk associated with traditional treatment methods like surgery or implantation. Once sprayed, the hydrogel rapidly forms a stable gel that closely conforms to the tumor surface. This provides a reliable platform for drug delivery and tumor treatment. For example, Ma et al. developed a sprayable β-FeSi2 hydrogel. The β-FeSi_2_ (FS) exhibited excellent photothermal performance, and the released Fe ions could generate •OH under acidic conditions and in the presence of excessive H_2_O_2_ in the tumor microenvironment. The sprayable β-FeSi2-incorporated sodium alginate (FS/SA) hydrogel, which instantaneously gels upon spraying, promotes timely tumor-induced skin wound healing and efficiently suppresses tumors through photothermal and chemodynamic therapy (PTT and CDT) [247]. Sprayable hydrogels can also be combined with immunotherapy to enhance the anti-tumor effect by regulating the tumor microenvironment and activating the immune system [248]. With the development of material science, biomedical engineering, and nanotechnology, the performance of sprayable hydrogels will be enhanced, leading to broader and more profound applications in tumor therapy.

### 6.5. Burn Wound Healing

Burn wound-healing is a complex process, and burns can cause serious damage to the skin, leading to pain, inflammation, bacterial infection, scarring, and even threatening the patient’s life. Thick, full-thickness burns require a bulky dressing; a thin sprayable layer alone is inadequate. The advantage of sprayable hydrogels is limited to superficial partial thickness burns where a thin, breathable, non-maceration film is needed. Superficial burns limited to the epidermis typically heal on their own within 7 days. For superficial second-degree burns involving the upper layer of the dermis, the standard healing time is approximately 14 days. When re-epithelialization takes longer than 21 days, the risk of developing hypertrophic scars increases significantly. Therefore, the current standard for burn care is to achieve complete epithelialization within 14 to 21 days. Burn wounds pose great challenges for conventional dressings because massive exudates secreted from swollen tissues and blisters seriously delay wound healing [249]. Hydrogel wound dressings have strong water-absorption properties, effectively absorbing blood, pus, and other fluids oozing from burn wounds. They help keep the wound dry and clean, reducing the risk of infection. Additionally, the moist environment provided by hydrogel wound dressings promotes cell proliferation and epithelial cell migration, accelerating the healing of burn wounds [250]. Liu et al. used Pluronic F127 (PF127, also known as Poloxam 407) combined with a coordination complex of zinc and metformin (ZnMet) to establish a new sprayable adhesive (ZnMet-PF127). Sprayable ZnMet-PF127 can evenly cover irregular skin defects in liquid form at room temperature or below and solidify into a semi-solid upon contact with the skin surface. The experiment demonstrated that in the ZnMet-PF127 group, the burn wound healing rate peaked on the ninth day, and by the seventeenth day, the wound was nearly completely closed. The hydrogel can effectively promote the healing of traumatic skin defects and burn injuries by enhancing cell proliferation, angiogenesis, and collagen formation [24]. In the first aid of burns, sprayable hydrogel wound dressings can be quickly applied to the wound to cool, moisturize, relieve pain, and prevent infection, creating favorable conditions for subsequent treatment. Moreover, they can be easily and quickly applied to burn wounds, especially for wounds with a large area and irregular shape.

In partial-thickness burns, the standard complication is maceration and delayed re-epithelialization when thick ointments or gels are applied. The acceptable threshold is complete healing within 14–21 days. A sprayable hydrogel forms a thin, semi-permeable film that maintains moisture without drowning the regenerating epidermis. This cannot be achieved with a topical syringe gel, which tends to form a thick, occlusive layer. Furthermore, sprayable hydrogels are ideal for fixing split-thickness skin grafts: a thin layer of sprayable adhesive provides uniform tack, eliminates the need for sutures, and allows the graft to take without shearing; again, a topical gel would be too thick and might displace the graft [251].

### 6.6. Joint Wound Healing

For a common skin wound, traditional treatments such as bandaging the wound with gauze are sufficient. Meanwhile, there are many significant problems when gauze is applied to joint injuries, for example, the wrist, ankle, and knee. A joint wound is subject to frequent movement during daily activities, leading to an unstable connection between the wound and the dressing. This instability increases the patient’s discomfort and places greater demands on wound healing [252]. Joint wounds are often irregular in shape, making it difficult to completely cover and secure them with traditional dressings. Moreover, due to the poor blood circulation in the joint area and its susceptibility to external contamination, joint wounds are prone to infections. Hydrogel dressings are considered ideal for wound care. However, most hydrogel wound dressings are better suited for skin wounds in flat areas, with few designed for skin wounds in joint areas that experience frequent movement. This mismatch in design between the hydrogel dressing and the skin wound in the joint can result in issues such as hydrogel loss, bacterial infections, and delayed healing [253]. Sprayable hydrogels can tightly adhere to irregular surfaces of joint wounds and maintain stable adhesion during joint movement, while also providing good elasticity and not restricting normal joint movement. Hu et al. developed a sprayable zwitterionic antibacterial hydrogel with high mechanical elasticity and strong adhesion for the treatment of joint wounds. The hydrogel achieves effective adhesion to irregular wound surfaces through the strong electrostatic interaction between zwitterionic sulfobetaine methacrylate (SBMA) and poly(sulfobetaine methacrylate-co-dopamine methylacrylamide)-modified silver nanoparticles (PSBDA@AgNPs). It also possesses properties such as high elasticity, resilience, adhesion, and self-healing. The experimental results indicate that the hydrogel has significant potential to enhance the healing of joint skin wounds [25]. Ding et al. developed a sprayable black phosphorus (BP)-based multifunctional hydrogel with on-demand removability for use as joint skin wound dressing. The hydrogel can conform well to the joint site, and it possesses excellent antibacterial and antioxidant properties. This sprayable BP hydrogel can effectively enhance joint wound healing by accelerating angiogenesis, reducing inflammation, and optimizing the wound microenvironment [254]. In the future, more hydrogel materials with excellent properties will be developed to meet the treatment needs of various types of joint wounds.

The desirable properties described above translate into distinct clinical scenarios with varying levels of maturity (Table 4). Rapid hemostasis and infection control in partial-thickness burns are already clinically feasible using oxidized cellulose or chitosan-based sprays. Diabetic foot ulcer management and skin graft fixation remain at the animal-model stage, hindered by chronic hypoxia, biofilm formation, and the need for on-demand removability. The retention of injectable scaffolds and smart sensing are currently speculative; key hurdles include displacement by wound exudate, long-term biocompatibility, and power supply for sensors.

## 7. Future Outlooks

**Figure 8:**
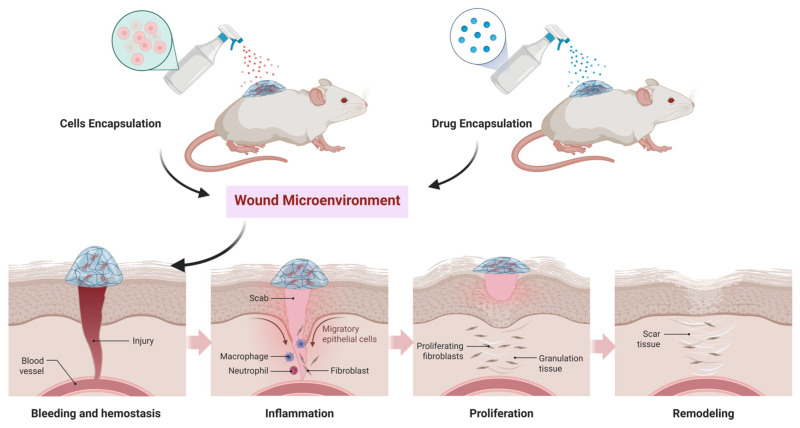
Sprayable hydrogel dressings to modulate the wound microenvironment in wound healing.

Although sprayable hydrogels can be used to deliver drugs and achieve passive drug release, the wound-healing microenvironment also needs to be considered during the wound-healing process (Figure 8). Future research can moderately incorporate responsive design in specific scenarios, such as chronic wounds, based on particular clinical needs. For example, in microenvironments characterized by abnormal pH levels or enzyme activity, systems capable of detecting relevant biomarkers and releasing therapeutic agents can be developed. It is important to note that for most ordinary wounds, the healing process can be completed through the body’s natural repair mechanisms following the initial application of wound dressings, and complex full-loop regulation is not always necessary. Enabling the Janus properties of sprayable hydrogels can solve various problems in clinical settings. Based on the specific type of the patient’s wound (e.g., diabetic ulcers, burns, and surgical trauma) and the healing stage, adjusting the components, mechanical properties, and release kinetics of the materials enables an optimal, personalized treatment plan for each patient. In addition, for deep cavity wounds, sinus tracts, or wounds with significant tissue defects, simple spraying is insufficient to achieve effective deep filling. In such cases, the film formed by spraying may only cover the wound surface and fail to penetrate the deeper areas of the defect, potentially leading to delayed healing or residual dead space. Therefore, in clinical practice, sprayable hydrogels should be used in combination with other application methods, such as injection filling with a syringe or implantation of preformed scaffolds.

The sprayable hydrogels should not only serve as wound coverings but also act as retention matrices for co-injected therapeutic scaffolds and cells. Additionally, the sprayable hydrogels in ultra-thin conformal coatings and skin graft fixation need to be further explored. Sprayable hydrogels could be fabricated via spray-assisted layer-by-layer assembly or handheld bioprinters, enabling conformal deposition of biocompatible polyelectrolytes onto wound beds. Then, the sprayable hydrogels could serve as temporary anchors for split-thickness skin grafts by promoting rapid adhesion, hemostasis, and cell viability. Simultaneously, these in situ forming hydrogel layers act as retention matrices for injectable micro-scaffolds, preventing their displacement and facilitating controlled release of therapeutic agents, ultimately enhancing graft integration and tissue regeneration [270,271,272]. Technologies for real-time wound monitoring (e.g., pH, temperature, and bacterial count via embedded sensors) and AI-assisted design of personalized dressings are rapidly evolving. It is important to note that these technologies are not exclusive to sprayable hydrogels; they can be integrated into injectable, pre-formed, or even conventional gauze-based systems. Therefore, while we acknowledge their potential, they are not the focus of this review. For sprayable hydrogels specifically, the most promising short-term developments lie in improving retention on exuding wounds (e.g., by incorporating mussel-inspired adhesives), enabling on-demand removability (e.g., using thermos-responsive or dissolvable crosslinkers), and combining sprayable top-layers with injectable fillers for cavity wounds—a strategy that is already feasible in animal models and deserves clinical translation. For some major hurdles still remain, including (i) detachment on exuding wounds due to weak wet adhesion, such as the sprayable hydrogels that could be improved by incorporating catechol or zwitterionic groups; (ii) non-uniform coverage caused by variable spraying distance and angle, and such issue could be solved by developing pressure-controlled nozzles; and (iii) inability to fill deep cavities; thus, a fundamental limitation requires combination with injectable fillers. Future designs need to more ingeniously utilize composite materials and dynamic cross-linking chemistry to achieve the optimal combination of properties. At the same time, smart sprayable dressings integrated with real-time monitoring functions, such as monitoring wound pH, temperature, or bacterial count via embedded sensors, will be an important prospect, and coupling these systems with AI-driven feedback loops could enable closed-loop therapeutic management.

## 8. Conclusions

Sprayable hydrogels, due to their unique minimally invasive application, strong adhesion to irregular wounds, and ability to deliver bioactive factors, have become a promising treatment method in wound management. Sprayable hydrogels have demonstrated clinical utility in the following three well-defined scenarios: (i) rapid hemostasis in external bleeding, (ii) infection control and moisture management in partial-thickness burns, and (iii) prevention of post-operative abdominal adhesions. The key enabling properties are now well-understood and can be achieved using a range of natural and synthetic polymers. This review systematically summarizes the natural and synthetic polymers used to prepare sprayable hydrogels, discusses key performance factors such as rheology, wet tissue adhesion, mechanical strength, and biodegradability, and elaborates on various crosslinking mechanisms for achieving in situ rapid gelation. By summarizing their applications across different types of wound healing, we can more clearly see that, through reasonable molecular design, sprayable hydrogels can effectively promote hemostasis, prevent infection, regulate inflammation, support cell proliferation and migration, and thereby comprehensively accelerate tissue regeneration. Technologies such as real-time sensors, AI-driven formulation, and closed-loop drug delivery are promising but are not unique to sprayable hydrogels. Their development should follow general wound dressing research and is not covered here. In summary, this review provides a clear separation between proven applications, problem-solving scenarios with adaptable solutions, and future hurdles requiring proof-of-concept. We hope this review paper helps researchers prioritize their efforts towards the most impactful next steps.

## Figures and Tables

**Table 1:** Comparison of crosslinking methods of sprayable hydrogels.

Crosslinking Strategy	Photopolymerization	Chemical Crosslinking	Physical Crosslinking
Advantage	Precise control	Formed spontaneously, no equipment required	High biocompatibility, non-toxic
Disadvantage	The light can penetrate only to a limited depth. Phototoxicity	The potential toxicity of chemical initiators	Weak mechanical properties
Cytotoxicity	High	High	Low

**Table 3:** Synthetic polymers used for fabricating sprayable hydrogel dressings.

Synthetic Polymer	Chemical Structure	Hydrogel Form	Types of Studies In Vitro/In Vivo	Main Properties	Ref.
Poly (ethylene glycol)	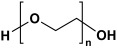	Gelation through solution blow molding technology with polylactic acid–glycolic acid copolymer.	Antimicrobial activity and L929 cytotoxicity, porcine wound model, and dermal and epidermal tissue regeneration.	The antibacterial effect is remarkable. It promotes tissue regeneration, reduces the frequency of dressing changes, has good biocompatibility, and maintains a moist environment around the wound.	[142,143]
Polyvinyl alcohol	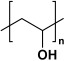	Cross-linked with alginate using borax and calcium chloride to form a gel.	In vitro degradation, antibacterial experiment, and rat full-thickness skin defect model.	Mechanical protection, accelerating tissue contraction, enhancing epithelialization, promoting granulation tissue proliferation, stimulating angiogenesis and collagen synthesis.	[144,145]
PNIPAm	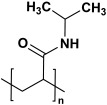	Thermosensitive gelation.	Cytotoxicity test, cell proliferation test, and adhesion of isolated porcine intestinal tissue.	Good biocompatibility, promoting cell proliferation and tissue regeneration, with uniform wound coverage.	[146,147]

**Table 4:** Current feasibility, remaining hurdles, and speculative uses of sprayable hydrogels in wound healing.

Applications	Already Proven (Clinical or Large-Animal)	Remaining Hurdles	Speculative/Needs Development	Ref
Rapid hemostasis in external bleeding	Yes (oxidized cellulose, chitosan sprays)	Adhesion on wet blood-covered surfaces	Sealing of high-pressure arterial bleeding	[255,256,257]
Partial-thickness burns	Yes (moisture-retentive, antibacterial sprays)	Controlling scar formation; painless removal	Smart release of anti-fibrotic agents	[258,259]
Diabetic foot ulcers	Animal models only	Chronic hypoxia, biofilm, and need for repeated application	Oxygen-generating sprays, pH-responsive drug release	[260,261,262]
Skin graft fixation	Animal models only	Ensuring uniform adhesion without graft shearing	Sprayable bio-glues with exactly matched degradation	[251,263]
Smart sensing (pH, temperature, and bacteria)	Not yet in any hydrogel system	Power supply, data transmission, and biocompatibility of sensors	Closed-loop therapeutic feedback (for all hydrogel types)	[264,265,266]
Retention of injectable scaffolds	Proof-of-concept in vitro	In vivo displacement by wound exudate; long-term safety	Combined sprayable top-layer + injectable filler	[267,268,269]

## Data Availability

Data will be made available on request.
